# The influence of hand depiction types on behavioural patterns in laterality judgments

**DOI:** 10.1371/journal.pone.0352694

**Published:** 2026-07-13

**Authors:** Aneet K. Saran, Stephen Wood, Jonathan J. Marotta

**Affiliations:** 1 Department of Psychology, University of Manitoba, Winnipeg, Manitoba, Canada; 2 Neuropsychology of Vision: Perception & Action Laboratory, University of Manitoba, Winnipeg, Manitoba, Canada; Gabriele d’Annunzio University of Chieti and Pescara: Universita degli Studi Gabriele d’Annunzio Chieti Pescara, ITALY

## Abstract

According to Motor Simulation Theory, cognitive states such as kinesthetic motor imagery activate the motor system in a manner similar to overt motor execution. Action simulation can be implicitly triggered when individuals unconsciously simulate an action, as in the *Hand Laterality Judgment Task* (HLJT). Studies employing the HLJT use different hand depictions, which may influence behavioural performance. The aim of the present study was to examine whether hand depiction type (realistic hands versus line drawings) influences performance on the HLJT. Sixty-two younger adults completed the HLJT using both realistic and line-drawing representations of hands. Results demonstrated significant effects of orientation, with faster and more accurate responses observed at less challenging orientations and a *medial-over-lateral advantage* (MOLA) evident for palm-view stimuli. Overall, performance declined as hand orientation became increasingly biomechanically demanding. Importantly, the effects of hand depiction type varied as a function of viewpoint and orientation. For back-view stimuli, line drawings elicited faster responses than realistic hands at 0° and 90° lateral orientations. In contrast, for palm-view stimuli, line drawings elicited faster responses and higher accuracy than realistic stimuli. These findings suggest that the effects of hand depiction are not uniform across task conditions. As such, the type of hand stimulus used should be carefully considered when designing, interpreting, and comparing HLJT studies, particularly when the task is used to assess motor imagery processes.

## Introduction

### Biomechanical constraints in hand laterality judgments

The ability to spatially transform a mental image can be implicitly triggered when individuals unconsciously simulate an action [1,2]. Implicit cognitive processes are also employed in the *Hand Laterality Judgement Task* (HLJT), in which participants are asked to determine whether a depicted hand presented at different angles is right or left hand [1]. It is thought that participants solve this task by mentally rotating their own hand into the orientation of the visually presented hand [3–6]. Parsons (1987) demonstrated that the time required to mentally rotate one’s hand closely mirrors the time required to physically execute the corresponding movement [1,3]. Unlike external objects, mental rotation of one’s own hand is constrained by biomechanical and motor rules that govern real movements [4,7].

These biomechanical constraints produce characteristic asymmetries in HLJT performance. Medial hand orientations (rotations toward the body midline) are biomechanically efficient and yield faster, more accurate responses. In contrast, lateral orientations (rotations away from the midline) require movements that approach or exceed the limits of joint mobility, resulting in longer response times and higher error rates [3,8,9]. This asymmetry gives rise to the medial-over-lateral advantage (MOLA; [3,9,10]). Notably, hands presented at 90° lateral orientations elicit slower responses than those at 180°, producing a non-linear response time pattern that cannot be explained by rotational distance alone but reflects the biomechanical difficulty of the imagined movement [3,8]. The MOLA has been observed across diverse populations, including individuals with congenital limb deficiencies and clinical groups such as those with Parkinson’s disease, chronic pain, and focal hand dystonia, although typically with attenuated performance [11–16]. While biomechanical constraints influence HLJT performance, other stimulus characteristics may also contribute to behavioural outcomes. One such characteristic is the viewpoint from which the hand is presented.

### Palm and back views in hand laterality judgments

Anatomical constraints are more pronounced for palm than back hand views, with palm views eliciting longer response times and greater errors for lateral compared to medial orientations, indicating stronger biomechanical effects [1,3,9,17–19]. Neuroimaging evidence suggests that palm and back views may differentially engage motor and visual processes [18]. This dissociation supports the proposal that palm views more consistently recruit motor-based strategies, whereas back-view stimuli may permit greater variability in strategy use, with participants relying on visual imagery, pattern matching, object-based mental rotation, or motor simulation depending on individual differences and task demands [17,19–23]. When motor imagery is employed for hand rotation, patients with parietal damage show selective impairments compared to external object rotation, reinforcing the distinct neural substrates underlying motor-based hand imagery [24,25].

Beyond viewpoint, limb laterality also influences hand mental rotation, consistent with the body-specific hypothesis [17,26,27]. Right-handed individuals typically recognize right-hand stimuli faster and show left-hemisphere dominance for motor processing, as supported by behavioral and electrophysiological findings [20,28–30]. Additionally, individuals may show a self-advantage when mentally rotating their own hands relative to others’, likely due to greater familiarity, body ownership, and reliance on egocentric reference frames [19,31–33]. This effect is supported by evidence that egocentric hand views elicit stronger activation in body-selective regions such as the extrastriate body area (EBA) [33,34]. Finally, proprioceptive information further constrains hand mental rotation. Changes in actual limb posture disrupt response times when imagining one’s own hands, but not when adopting a third-person perspective, indicating that motor imagery of hands is governed by the same biomechanical constraints as real actions [17]. Together, these findings highlight the influence of body-specific factors on performance during hand laterality judgments.

### The current study

When employing the *Hand Laterality Judgement Task* (HLJT), studies tend to use different types of hand stimuli, such as line drawings or realistic hands [5,28,29,35–37] to investigate explicit and implicit motor imagery [1,3]. Although different hand depiction types are commonly used in the HLJT, relatively little is known about how stimulus depiction influences performance [38]. Given that stimulus format varies considerably across studies, understanding its effects is important for interpreting existing findings and selecting appropriate stimuli for future research and clinical applications.

Prior research suggests that embodiment and ownership may influence motor imagery processes [19,31,39]. Consistent with this, realistic hand stimuli have been shown to induce greater embodiment than mechanical hand stimuli in the rubber hand illusion [40]. As realistic hand depictions contain richer anatomical information than schematic line drawings, they may differentially influence behavioural performance during hand laterality judgments.

The present study examined whether hand depiction type influences behavioural performance during the HLJT and whether these effects differ between palm and back views. Given evidence that palm and back views are associated with distinct behavioural and neural patterns, these views were examined separately to determine whether depiction-related effects differed across viewing conditions. Consistent with previous research, we expected performance to vary as a function of hand orientation, with evidence of a medial-over-lateral advantage (MOLA) for palm-view stimuli [3,9,10]. We further hypothesized that behavioural performance would differ between realistic and line-drawing hand stimuli.

## Methods

Participants completed a *Hand Laterality Judgment Task* (HLJT) in which they assessed the laterality of hand stimuli presented across different orientations, views, and depiction types.

### Participants

Seventy young adults were recruited from the University of Manitoba’s psychology participant research pool and received 1 experimental credit for participation. Eight participants were excluded due to error rates exceeding 30%, resulting in a final sample of 62 participants (40 female, 22 males; age range = 16–37 years, M = 19.92, SD = 3.98) included in all analyses. A power analysis using G*Power version 3.1 was conducted [41]. To achieve a power of 0.80 and detect a large effect size 0.25 at a significance level of α = .05, the analysis suggested a total of 20 participants for a repeated measures Analysis of Variance (ANOVA). Each participant provided informed consent before beginning the experiment. Participants completed several demographic questions regarding age, sex, visual acuity, and handedness (using a modified version of the Edinburgh Handedness Inventory; [42]). All participants had normal or corrected to normal vision and were right- hand dominant as determined by a modified version of the Edinburgh Handedness Inventory. All participants engaged in regular physical exercise, cognitive activities, (e.g., reading books, doing puzzles, etc.) and had no known neurological problems. On average, younger adults participated in physical and cognitive activities for 4 days a week. After the testing session, all subjects underwent debriefing. The research complies with the American Psychological Association ethical standards in the treatment of participants and has been approved by the Research Ethics Board at the University of Manitoba, Fort Garry campus.

### Stimuli and materials

**Fig 1:**
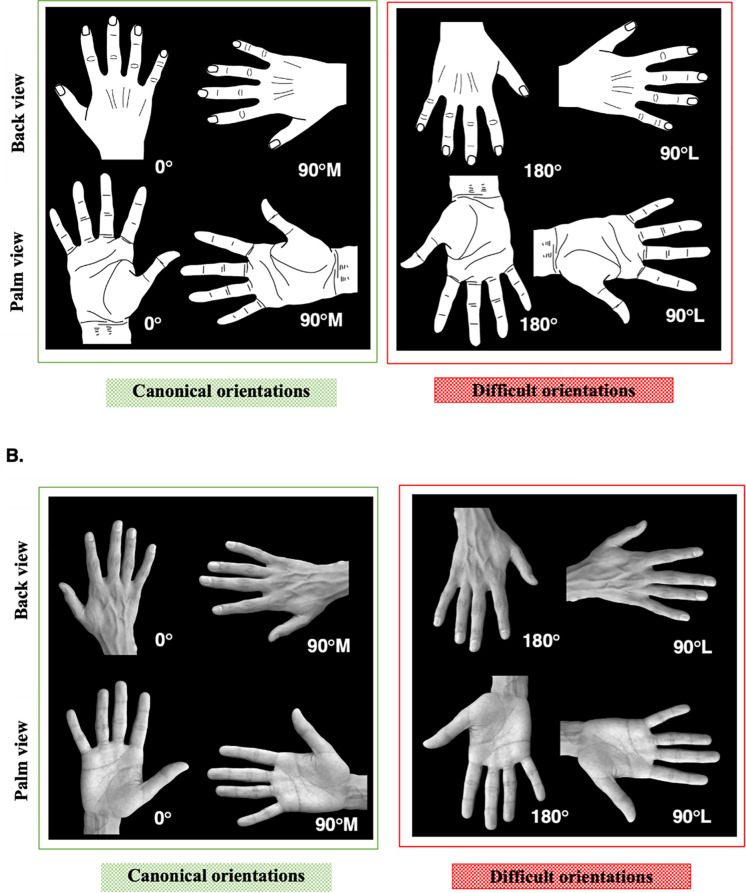
Right-hand stimuli used in the task. Examples of line drawing (top) and real hand (bottom) stimuli shown from palm and back views at four orientations: 0°, 90° medial, 90° lateral, and 180°.

The task was created using lab.js, an open-source online experimental platform for behavioral and cognitive sciences [43]. The experimental stimuli consisted of either line drawings or photographs of left and right hands. The realistic stimuli consisted of grayscale photographs presented on a black background, whereas the line-drawing stimuli consisted of simplified two-dimensional representations matched for laterality, viewpoint, and orientation. All participants completed the task in-lab using the same monitor and display settings. Realistic and line-drawing stimuli were scaled to occupy comparable areas of the display. These stimuli were displayed from two different viewpoints (back and palm) and in four different orientations: 0° = upward position, 90° medial = facing toward the midsagittal plane of the body, 90° lateral = facing away from the midsagittal plane of the body, and 180° = downward position (Fig 1). Orientations were selected based on previous research where rotation angles were increased by 90° [1,3,17,36]. The trials were interspersed with a white fixation cross, and a target image remained on the screen until the participants indicated the laterality by either pressing either the left or right button on the keyboard. The 192 experimental trials comprised 6 repetitions of each unique stimulus combination (2 laterality × 4 orientations × 2 depiction types = 32 unique conditions), with trials presented in randomized order. The task was programmed in lab.js and hosted on the online platform GitHub.

### Procedure

Prior to beginning the experiment, participants were instructed to complete a variety of demographic questions followed by the SRT task. The SRT task was included as a measure of baseline motor responding to account for individual differences in general motor speed that could potentially influence HLJT performance. In the SRT task, participants were instructed to focus on a fixation cross (displayed for 1500 ms) and press the space key with their right or left hand when they saw a red circle. Twenty trials were performed for each hand. Participants completed two training phases to familiarize themselves with the experimental protocol. Similar familiarization procedures have been used in previous HLJT research [44,45]. In training phase one, 32 unique hand stimuli were presented on the screen to ensure all participants could physically move and match their hands to the stimuli. Participants pressed the ‘Y’ key to indicate their ability to physically move and match their hand to the image displayed on the screen, and the ‘N’ key if they are unable to do so. Each trial began with a white fixation cross appearing on the computer screen for 1500ms, followed by a target hand image that remained on the screen until the participants physically attempted to move and match their hand to the orientation and view of the hand-stimuli.

The second training phase was designed to familiarize participants with left-and-right hand images presented in different orientations and views. In this training phase, participants were asked to rest their hands palm-down on the keyboard, so that their right index finger was positioned above the ‘K’ key, and their left index finger was positioned above the ‘A’ key. Participants completed 32 practice trials in this training phase to ensure they are performing the trials according to the instructions. Participants were instructed not to physically move and match their hands to the image displayed on the screen. Instead, participants selected the ‘K’ key for right hand-stimuli and the ‘A’ key for left hand-stimuli. The trials were interspersed with a white fixation cross (displayed for 1500 ms), and the target image remained on the screen until the participants indicated the laterality by either pressing either the left or right button on the keyboard. Once the trial ended, participants were shown a screen with a continue prompt to prepare them for the next trial. Once both training phases were complete, participants began the experimental phase. The experimental phase included the same instructions provided in phase two, except participants were instructed to respond as quickly and accurately as possible, reflecting a balanced speed-accuracy emphasis. No explicit guidance was provided regarding strategy use (e.g., whether to imagine moving one’s own hand or to treat the stimulus as an external object). Upon finishing the experiment, participants were directed to the debriefing form.

### Data processing and analysis

The present study examined whether hand depiction type influences behavioural performance during hand laterality judgments. Participants determined the laterality of right and left hands from different views and orientations using line depictions and realistic hands. The independent variables were hand laterality (right vs. left), view (back vs. palm), orientation (0°, 90° medial, 180°, 90° lateral), and stimulus type (line depictions vs. realistic hands). Dependent measures were response time (RT), defined as the interval between stimulus onset and button press, and accuracy, defined as the number of correct responses coded trial-by-trial (1 = correct, 0 = incorrect; missing = NA).

### Data exclusion

Trials with durations shorter than 500 ms or longer than 7500 ms were removed. Trials with incorrect laterality judgments were additionally excluded from response time analyses.

These criteria were applied to exclude anticipatory responses and excessively delayed responses that were unlikely to reflect task-related processing, consistent with previous HLJT research [44]. Overall, 10.22% of trials were removed from the final analyses.

### Statistical analyses

Trial data within each condition were averaged to create mean condition values for each participant. Response times for correct trials were analyzed separately for palm- and back-view stimuli using repeated-measures ANOVAs with factors of laterality (right vs. left), orientation (0°, 90° medial, 180°, and 90° lateral), and stimulus type (line drawing vs. realistic hand) in jamovi (Version 1.6). Violations of sphericity were assessed using Mauchly’s test and addressed with Greenhouse–Geisser corrections.

Accuracy data were coded as binary outcomes (0 = incorrect, 1 = correct), which violate the distributional assumptions required for ANOVA. Accordingly, accuracy was analyzed using a generalized linear mixed model (GLMM) with a binomial distribution and logit link function for palm-and back-view stimuli in jamovi (Version 1.6), with laterality, orientation, and stimulus type as fixed effects and participant ID as a random effect. Bonferroni-corrected pairwise comparisons were conducted when interactions were present (p < .05).

## Results

### Simple reaction time (SRT)

Simple reaction time (SRT) was also measured to index baseline motor responding. The average SRT was 245.12 ms (SD = 65.29) for right-hand responses and 264.85 ms (SD = 115.34) for left-hand responses.

### Response times

#### Back view.

A significant main effect of Hand Type was observed, *F*(1, 61) = 35.76, *p* < .001, ηp² = .370, with participants responding significantly faster to line-drawing stimuli than realistic hands. A significant main effect of Orientation was also found, *F*(3, 183) = 109.90, *p* < .001, ηp² = .643.

**Fig 2:**
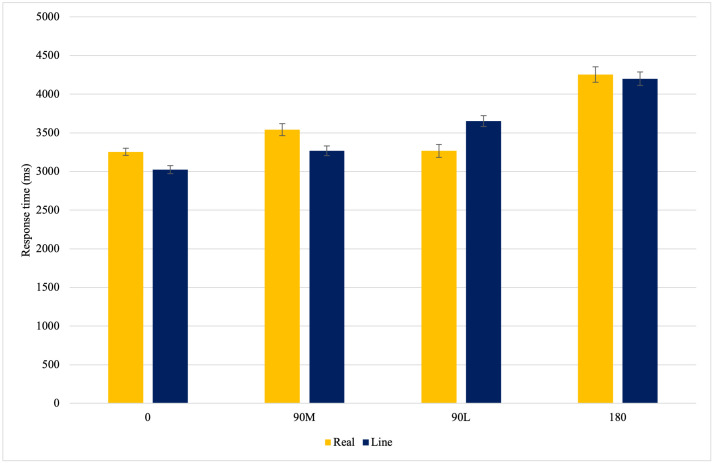
Mean response times for realistic and line-drawing stimuli across hand orientations. Error bars represent standard errors.

Response times increased as orientation became more difficult, with the fastest responses observed at 0° and the slowest responses observed at 180°. A significant Hand Type × Orientation interaction was found, *F*(3, 183) = 3.49, *p* = .017, η² = .054 (Fig 2). When viewing the back of the hand, response times increased as orientation became more difficult for both realistic and line-drawing stimuli. Participants responded significantly faster to line-drawing than realistic hand stimuli at 0° (*p* < .001) and 90° L (*p* = .003). In contrast, no differences between depiction types were observed at 90° M or 180°.

#### Palm view.

**Fig 3:**
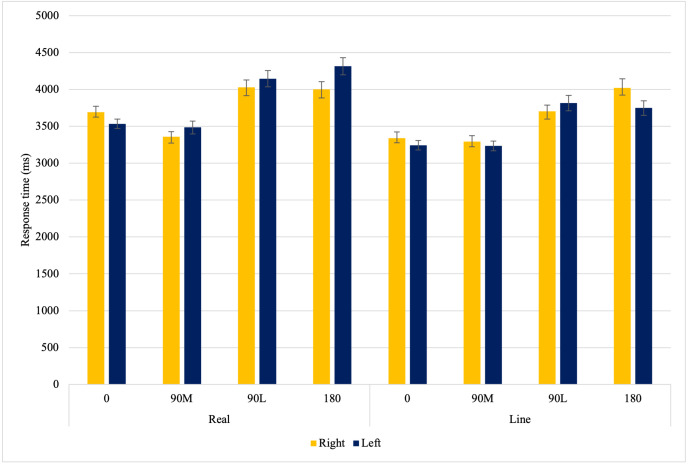
Mean response times for right- and left-hand stimuli across orientations and depiction types (realistic and line drawings). Error bars represent standard errors.

A significant main effect of Hand Type was observed, *F*(1, 61) = 69.7761, *p* < .001, ηp² = .534, with participants responding significantly faster to line-drawing compared to realistic hand stimuli. A significant main effect of Orientation was also found, *F*(3, 183) = 56.61, *p* < .001, ηp² = .481. Response times increased as orientation became more difficult. Notably, participants responded significantly faster at 90° M than at 0°, 90° L and 180° orientations. Responses were also significantly faster at 0° than at 90° lateral and 180° orientations. A significant Hand Type × Laterality interaction was observed, *F*(1, 61) = 10.28, *p* = .002, η² = .144. Faster response times were observed for line-drawing compared to realistic stimuli for both right- and left-hand stimuli (*ps* < .001). A significant Hand Type × Laterality × Orientation interaction was observed, *F*(3, 183) = 6.16, *p* < .001, η² = .092 (Fig 3). Realistic stimuli elicited the fastest responses at 90° M orientations, whereas line-drawing stimuli elicited the fastest responses at both 0° and 90° M orientations. For both stimulus types, responses at 90° L and 180° orientations were significantly slower than the fastest orientations (*ps* < .001).

### Accuracy

#### Back view.

**Fig 4:**
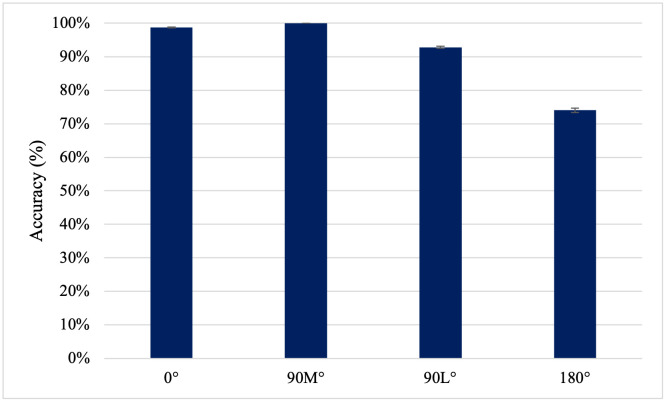
Mean accuracy rates across hand orientations. Error bars represent standard errors.

A significant main effect of Orientation was observed for back-view stimuli, χ²(3) = 14.11, *p* = .003, with lower accuracy observed at 180° compared to 0° (*p* = .002; see Fig 4).

#### Palm view.

**Fig 5:**
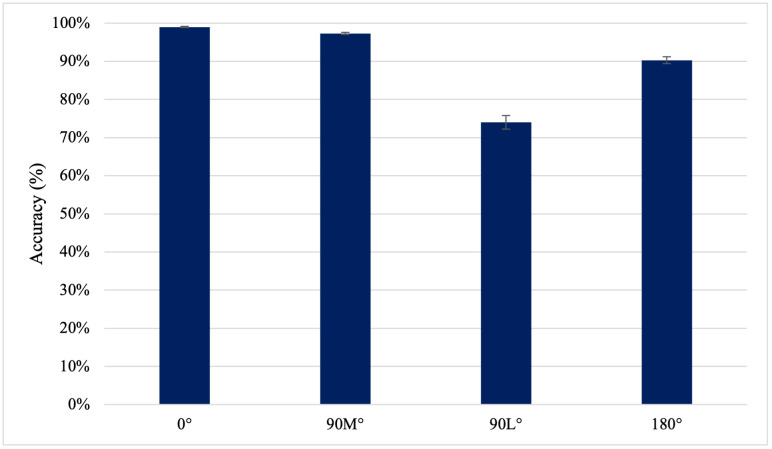
Mean accuracy rates across hand orientations. Error bars represent standard errors.

**Fig 6:**
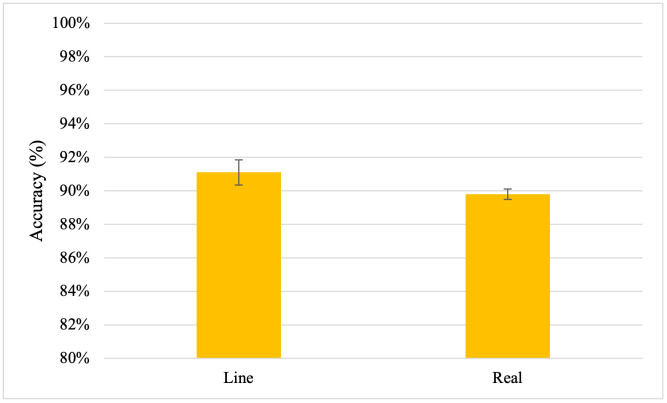
Mean accuracy rates for line-drawing and realistic stimuli. Error bars represent standard errors.

A significant main effect of Orientation was observed for palm-view stimuli, χ²(3) = 24.29, *p* < .001, with lower accuracy observed at 90° lateral (*p* < .001) and 180° (*p* = .005) compared to 0°, and 90° medial orientations (Fig 5). A significant main effect of Hand Type was also observed, χ²(1) = 5.29, *p* = .022, with higher accuracy observed for line-drawing stimuli than realistic stimuli (Fig 6)

## Discussion

The present study examined whether hand depiction type (realistic vs. line drawings) influenced one’s performance on the *Hand Laterality Judgment Task* (HLJT). For back-view stimuli, line drawings elicited faster responses than realistic stimuli at 0° and 90° lateral orientations. For palm-view stimuli, line drawings elicited faster responses and higher accuracy than realistic stimuli. In addition, participants responded faster and more accurately at canonical orientations than at more difficult orientations, and a *medial-over-lateral advantage* (MOLA) was observed for palm-view stimuli. Overall, performance declined as hand orientation became increasingly biomechanically challenging. This pattern is consistent with previous research suggesting that covert motor actions mirror the constraints of overt motor execution. The relatively small differences observed in SRT suggest that the reported HLJT effects are unlikely to reflect general differences in baseline motor speed. Taken together, these findings indicate that both hand depiction type and orientation influence behavioural performance during hand laterality judgments.

### Orientation effects and biomechanical constraints

When mentally simulating hand movements, response times tend to increase with angular disparity, mirroring the demands of physically rotating the hand [1,3,45–47]. Consistent with previous research, orientation significantly influenced performance for back-view stimuli, where response time increased with angular disparity [1,3,17]. Response times were fastest at 0° and progressively increased as orientation became more difficult, with the slowest responses observed at 180°. When viewing back of the hand, participants responded faster to hands rotated 90°M and 90°L than to those rotated 180°. Accuracy was also lower at 180° than at 0°, further indicating that more challenging orientations were associated with reduced performance.

For palm-view stimuli, response times did not increase linearly with the angle of the hand’s rotation. Participants responded fastest to 90°M orientations, even relative to 0° orientations, with slower responses observed for 90° L and 180° orientations. This pattern is consistent with the *medial-over-lateral advantage* (MOLA), whereby postures that are easier to achieve biomechanically are judged more quickly than those that are more difficult to achieve [1,10,35]. Accuracy analyses further supported this, with lower accuracy observed at 90° L and 180° orientations relative to 0° and 90°M orientations.

Taken together, these findings are consistent with the contribution of biomechanical factors to performance for palm-view stimuli, with postures that are more difficult to physically achieve resulting in slower and less accurate responses. It should be noted, however, that the use of motor imagery during the HLJT may not be universal. Mibu and colleagues (2020) found that at most 50% of participants demonstrated both the expected angle-dependent response time pattern and the biomechanical constraints effect in the palm-view condition, suggesting that a motor imagery-based strategy may not be adopted by all individuals. Nevertheless, the present findings are broadly consistent with the view that biomechanical constraints influence performance during hand laterality judgments, particularly for palm-view stimuli [10,35,48,49].

### Influence of hand depiction type

To the best of our knowledge, only one study has examined the differential effects of hand stimulus type used during the HLJT [38]. Moreno-Verdú and colleagues found that all stimulus types produced a biomechanical effect, although the magnitude of this effect differed across stimulus types, with realistic stimuli producing a larger biomechanical effect for palm-view hands.

These findings suggest that the visual characteristics of hand stimuli may influence performance during hand laterality judgments. Previous research has also associated embodied representations of the hand with greater recruitment of premotor and somatosensory regions [18]. The present study extends this work by comparing realistic hand photographs with line drawings. Whereas artificial hands retain three-dimensional structure, line drawings reduce the stimulus to two-dimensional contour and structural information, removing surface-level detail such as skin texture, shading, and colour. Despite this reduction, biomechanical effects were observed for both depiction types, indicating that biomechanical constraints were evident even when stimuli were reduced to simplified line drawings [36].

For back-view stimuli, line drawings elicited faster responses than realistic stimuli at 0° and 90° lateral orientations, whereas no differences between depiction types were observed at 90° medial or 180°. The line-drawing advantage at easier orientations may reflect the benefit of reduced visual complexity when the cognitive demands of mental rotation are relatively low. The dorsal surface of the hand is frequently viewed during everyday activities such as reaching, grasping, and gesturing, and is therefore associated with a rich stored visual representation [17]. Although realistic depictions may provide additional anatomical detail, this information may not necessarily facilitate performance and could instead introduce perceptual information that is not required for determining hand laterality [38]. At more challenging orientations, however, the demands associated with mentally transforming the hand may reduce the influence of surface-level visual features. This interpretation is consistent with evidence that more difficult hand rotations place greater demands on motor imagery processes [1,3], potentially diminishing any advantage conferred by simplified visual representations.

For palm-view stimuli, a different pattern emerged. Participants responded more quickly to line drawings than realistic hands, and higher accuracy was also observed for line-drawing stimuli. One possible explanation is that realistic hand stimuli contain greater visual and anatomical detail than line drawings, increasing the amount of information that must be processed before a laterality judgment can be made. This is consistent with previous research indicating that stimulus visual properties modulate processing demands during hand mental rotation [8,29]. The palm is viewed less frequently in daily life than the dorsal surface, and its visual representation may be less detailed [17]. For palm-view stimuli, the additional anatomical detail present in realistic images may therefore not confer a familiarity advantage and may instead introduce perceptual information that increases processing demands without aiding the laterality judgment. Line drawings, by stripping away this extraneous detail, may allow more efficient extraction of structural cues such as finger configuration and thumb position that are necessary for determining hand laterality [36]. Furthermore, previous research has shown that realistic hand stimuli produced larger biomechanical effects than other stimulus types, suggesting that increased realism may influence how biomechanical constraints are expressed during laterality judgments [38]. Taken together, these findings suggest that for palm-view stimuli, greater stimulus realism does not facilitate performance and may instead increase perceptual processing demands, resulting in slower and less accurate responses.

A few limitations should be considered when interpreting the results of this study. The present study was conducted in a healthy sample, limiting conclusions about how depiction type may influence HLJT performance in clinical populations. Future studies may explore whether HLJT performance differs in individuals with neurological, motor, or pain-related conditions, where simulation processes may already be disrupted. In addition, only two levels of stimulus realism were examined, which does not capture the full range of visual abstraction that may affect motor imagery processes. Additionally, luminance and contrast were not formally equated across depiction types. While this reflects the naturalistic differences between realistic photographs and line drawings that the study aimed to examine, future research may benefit from systematically controlling low-level visual properties to isolate their contribution to depiction type effects. A further limitation concerns the first training, during which participants were asked to physically match their hand to the orientation of the presented stimulus. Although this procedure was included to familiarize participants with the task, it may have increased awareness of the biomechanical constraints associated with the stimuli and encouraged the use of motor imagery during the subsequent HLJT. Despite these limitations, the present study has important strengths. One strength of the present study is that it is, to our knowledge, the first to directly compare realistic hand depictions and line drawings within the HLJT. In light of the widespread use of the HLJT, the current study enabled a more precise examination of how visual detail influences performance during laterality judgments.

## Conclusion

The *Hand Laterality Judgement Task* (HLJT) is a widely used tool for investigating mental simulations of hands and the influence of biomechanical constraints on laterality judgments. To date, only one study has looked at the effects of the stimuli employed in the task. The present study addressed this gap by comparing realistic hand photographs with line drawings across different views and orientations [38]. The results demonstrated that hand depiction type influenced response time and accuracy performance, although the nature of these effects differed across palm- and back-view stimuli. For back-view stimuli, line drawings elicited faster responses than realistic hands at the less demanding orientations, whereas for palm-view stimuli, line drawings elicited both faster responses and higher accuracy than realistic stimuli. Altogether, the present findings suggest that stimulus depiction is not a neutral design choice in the HLJT and can meaningfully shape behavioural outcomes.
