# Supplementary material for: The influence of hand depiction types on behavioural patterns in laterality judgments
Source: PLoS One. 2026 Jul 13;21(7):e0352694. doi: 10.1371/journal.pone.0352694 (PMC13362083; doi:10.1371/journal.pone.0352694)
Supplement: Attachment — Submitted filename: RevisionLetter.docx [file pone.0352694.s002.docx]

Dear Dr. Giulia Prete,

Thank you for the opportunity to revise our manuscript entitled “The Influence of Hand Depiction Types on Behavioural Patterns in Laterality Judgments”. We thank the reviewers for their thoughtful **comments and helpful suggestions.** Based on the suggested edits, we believe the manuscript has been significantly improved.

**In response to each comment, please find our responses below.** Within the edited document, we have referenced specific sections and page numbers where related edits (tracked changes) have been made.

**Reviewer 1:**

1. **Abstract: The aim of the study should be stated on the described background. Also a brief discussion of the results and of its scientific and clinical worth should be provided.**

- We thank the reviewer for this helpful suggestion. The Abstract has been revised to explicitly state the aim of the study within the theoretical background.

2. **Introduction:49-78: This paragraph can and should be shortened to the most important information required to understand the aim of the study. 79-150: Though the provided information is very interesting it is not clear how it supports the study and the rest of the article, especially regarding the detailed explanations. Please shorten this paragraph as well in a clear and supportive way.151-192: Here the proplem definition is provided and it should be stated such as. Skip the term hypothesis as it is not a thesis but a scientific article. Provide the aim of the study with 3 different aims in a more compact and shortened way.**

- The Introduction has been revised for clarity and conciseness. The paragraphs in lines 49-78 and 79-150 have been shortened to retain only information necessary to motivate the study. In addition, Hypotheses 1 and 2 have been reframed as established background phenomena rather than formal hypotheses. The section in lines 151–192 has been revised to present the problem definition and study aims, with the study-specific prediction previously described in Hypothesis 3 retained as the primary aim.

3. **Methods: 194-198: Provide the information to statistics at the end of the method section. 214-216: No results in the method section! 199-219: Please provide exclusion criteria.** 2**60-291: Shorten this paragraph, it is not a thesis. See comment above to 194-198.**

- The Methods section has been revised accordingly. Specifically, statistical information originally presented in lines 194-198 has been moved to the end of the Methods section. Text describing results has been removed from lines 214–216 to ensure that no results are reported within the Methods. Upon further inspection of the exclusion criteria, eight participants were removed from the original sample of 70. Exclusion criteria have now been clearly specified. Finally, the Data Processing and Analysis subsection (lines 260–291) has been shortened to remove thesis-style details.

4. **Results: Please be clear with the term “more errors” – this is not very accurate.**

- The phrase “more errors” has been replaced with more precise accuracy-based wording, while the statistical results and interpretation remain unchanged.

5. **Discussion: There is quite an extended interpretation of the results without further ideas regarding scientific purpose or clinical purpose. Please try to find the following structure for your discussion (Key findings, your results in the context of previous works, limitations, strengths, and ways to move the field further)**

- We have revised the Discussion to reduce extended interpretation and to more clearly reflect the scientific and clinical purpose of the study.
- We also added concise text describing the study’s clinical relevance and future research directions, clarified the role of stimulus realism in shaping HLJT performance and incorporated a brief limitations paragraph addressing sample characteristics and stimulus abstraction (see lines 547-561).

6. **Summary: Try to be more consistent and keep the information to a relevant amount of content. Though many of this information is interesting to you, be aware of the reader, and how much does it need to understand your aim of the study and the interpretation of your results. It might be interesting if the authors have any clinical ideas (see discussion point ways to move the field further) – so far the relevance of their results remain sparse, especially beyond the field of testing laterality.**

- We revised the Discussion to improve consistency, reduce non-essential detail, and keep the content closely aligned with the study aim and key findings.

**Reviewer 2:**

**Reviewer #2: General Comments**
This manuscript addresses an interesting question regarding the role of hand depiction type in the hand laterality judgment task. However, I have serious concerns about the transparency and structure of the Methods and Results sections. Essential details regarding data preprocessing and analytical decision rules are missing, and the hierarchical logic required to interpret effects in a four-factor design is not clearly respected. In particular, the outcome of the highest-order (four-way) interaction is not reported, which makes it difficult to evaluate the validity of the reported lower-order effects. Because these methodological and analytical issues substantially limit the interpretability of the results, I have not commented in detail on the Discussion section. I believe that the Methods and Results require substantial revision before the theoretical implications of the findings can be meaningfully assessed.

Major Comments

1. **[Introduction - Kinesthetic Motor vs. Visual Motor Strategy] The dichotomous classification of imagery strategies into kinesthetic (first-person) for palm views and visual (third-person) for back views appears overly simplified. Previous studies have suggested that responses to back-view stimuli may rely not only on visual motor imagery, but also on pattern matching or object-like mental rotation processes, similar to those used for non-body objects. While I agree that kinesthetic motor imagery is likely dominant for palm-view stimuli, the strategy employed for back-view stimuli may be more heterogeneous than described here. We therefore suggest that this section be substantially revised to reflect a more nuanced view of imagery strategies, and to more carefully integrate relevant prior literature.**

- We thank the reviewer for this insightful comment and agree that our original characterization of imagery strategies was overly dichotomous. We have substantially revised this section to reflect a more nuanced view of strategy heterogeneity, particularly for back-view stimuli (see lines 73-121).

2. **[Introduction - The Current Study] While the Introduction provides a comprehensive overview of biomechanical constraints and imagery strategies in the HLJT, the rationale for focusing on hand depiction type is not sufficiently developed. Given the strong emphasis on embodied and motor-based processing throughout the Introduction, it would strengthen the manuscript to more explicitly explain why differences in stimulus realism are theoretically relevant, and how hand depiction type relates to the previously discussed mechanisms. Clarifying this link would make the motivation for the current study more compelling.**

- We agree that the rationale for focusing on hand depiction type was not sufficiently developed in relation to the embodied and motor-based mechanisms discussed earlier in the Introduction. We have added this into the current study rationale.

3. **[Introduction - Hypotheses] Hypotheses 1 and 2 largely restate well-established phenomena in the HLJT literature, including canonical orientation effects and the medial-over-lateral advantage (MOLA), rather than articulating hypotheses that advance or differentiate the aims of the present study. As such, these statements read more like background assumptions than hypotheses and should be reconsidered accordingly. Hypothesis 3 is closer to a study-specific prediction than Hypotheses 1 and 2; however, it currently conflates hypotheses with explanatory statements and general assumptions about motor rules. The predicted direction of the reaction time effect (slower responses for realistic hands) is not sufficiently justified, the rationale for preserved accuracy remains unclear, and view-dependent differences emphasized earlier in the Introduction are not adequately accounted for. The authors should substantially refine Hypothesis 3 to focus on a clear, falsifiable prediction directly tied to hand depiction type.**

- We agree that Hypotheses 1 and 2 restated well-established phenomena rather than advancing study-specific aims, and that Hypothesis 3 required refinement. We have substantially revised this section accordingly (see lines 153-167)

4. **[Methods - Data analysis] The description of data handling and statistical analysis lacks essential methodological detail. First, the manuscript does not specify how incorrect responses and extreme RT outliers were treated, despite these procedures being critical for RT-based analyses. Second, reaction time and accuracy were analyzed using different statistical frameworks (repeated-measures ANOVA vs. GLMM), but the rationale for this discrepancy is not explained. Third, the analytical strategy for handling interactions is insufficiently specified; it remains unclear how the presence or absence of interactions guided subsequent analyses and interpretation (e.g., whether and how simple effects were examined). Explicitly defining these procedures is necessary to ensure transparency, reproducibility, and interpretability of the results.**

- This section has been revised to clarify data handling and statistical procedures.
- We also clarified the rationale for using different analytical frameworks for response time and accuracy.

5. **[Results – Response times] Reaction time and accuracy were analyzed using multi-factor models including Laterality, View, Orientation, and Hand Type. In such a four-factor design, I believe that the interpretation of effects should follow a clear hierarchical structure, beginning with the highest-order interaction. Specifically, the presence or absence of a four-way interaction (Laterality × View × Orientation × Hand Type) determines whether lower-order interactions and main effects can be meaningfully interpreted as general effects or only as conditional effects.**
   **In the current Results sections for both RT and accuracy, the outcome of the four-way interaction is not reported. Instead, selected three-way and two-way interactions are described, followed by unqualified main effects. Without explicitly stating whether the four-way interaction was tested and whether it was significant, I find it difficult to evaluate the scope and validity of the reported lower-order effects. Moreover, multiple three-way interactions are possible in this design, yet only a subset is reported, and the criteria for selecting and decomposing these interactions are not clearly specified. When higher-order interactions are significant, it is standard practice to either (i) focus interpretation on those higher-order effects and their planned decompositions (e.g., via simple-effects analyses), or (ii) explicitly justify why certain lower-order effects are examined independently. I therefore recommend that the authors (1) explicitly report the results of the four-way interaction for both RT and accuracy, (2) clarify a priori decision rules for proceeding from four-way to three-way and lower-order analyses, and (3) restructure the Results sections to reflect this hierarchical logic. Doing so would substantially improve the transparency, interpretability, and reproducibility of the findings.**

- We agree that the interpretation of effects in a four-factor design should follow a clear hierarchical structure, and we have substantially revised both the Methods and Results sections to address these concerns. We now explicitly report the outcome of the four-way interaction for response time. The four-way interaction for accuracy was not significant. Regarding a priori decision rules we have added an explanation to the data processing section (see lines 276-287).

**Minor Comments**

6. **[Abstract] The abstract is necessarily concise and generally reflects the main findings; however, it somewhat underrepresents the hypothesis-driven nature of the study and the role of accuracy analyses.**

- The abstract has been revised to more clearly reflect the hypothesis-driven nature of the study and to incorporate accuracy analyses more explicitly.

7. **[Abstract] While the abstract is necessarily concise, the use of abbreviations such as “90°L” may not be self-explanatory to a broad readership. A brief clarification (e.g., lateral orientation) would improve the clarity of the abstract.**

- We have replaced “90°L” with “90° lateral orientation” in the abstract to improve clarity for a broad readership.

8. **[Introduction - 1st paragraph] In the introductory description of the HLJT, the authors cite a preprint reference (Ref. 14). Given that this passage serves as a general definition of the task, it may be more appropriate to cite a canonical or review paper on the HLJT.**

- We agree that a canonical reference is more appropriate for the general definition of the HLJT. We have replaced the preprint reference (Ref. 14) with Parsons (1987), which is widely recognized as the foundational work establishing the hand laterality judgment paradigm.

9. **[Introduction - 1st paragraph] The explanatory section from the push-up example through the introduction of MOLA would benefit from substantial clarification. In its current form, the discussion mixes geometric notions of rotation with biomechanical constraints, which makes the underlying rationale difficult to follow. Reorganizing this section to emphasize biomechanical asymmetries as the core explanation for MOLA would improve conceptual clarity.**

- We agree that the original text conflated geometric notions of rotation with biomechanical constraints, which obscured the rationale for MOLA. We have substantially revised this section to emphasize biomechanical asymmetries as the core explanation.

10. **[Introduction - 1st paragraph] Ref. 15 may concern a foot laterality task; its use to support arm/shoulder pain may require clarification.**

- The reviewer is correct that Ref. 15 (Coslett et al., 2010) employed a foot laterality task rather than a hand laterality task. We have removed this reference from the sentence supporting hand mental rotation findings in clinical populations to ensure accuracy [see line 64-72].

11. **[Methods - 1st paragraph] The opening paragraph of the Methods section describes the experimental design using an ANOVA framework. While the statistical analysis itself is explained later, presenting the design primarily in terms of the analysis may blur the distinction between experimental design and statistical procedures. Clarifying the design independently of the ANOVA terminology (e.g., explicitly framing it as a factorial design) would improve conceptual clarity for the reader.**

- . We have revised the opening paragraph of the Methods section to describe the experimental task and design independently of the statistical analysis framework.

12. **[Methods - Participants] The simple reaction time (SRT) is reported, but it does not appear to be incorporated into subsequent analyses. Some prior HLJT studies adjust for individual differences in basic motor speed (e.g., by subtracting or otherwise controlling for simple RT). Please clarify the intended role of the SRT measure in the present study (descriptive only vs. used for adjustment), and, if not used, briefly justify why (and/or consider reporting analyses with motor-speed adjustment as a robustness check).**

- Simple reaction time (SRT) was collected to index baseline motor responding for descriptive purposes. We have clarified in the revised manuscript that SRT was not used to adjust HLJT response times, as the within-subjects design inherently controls for individual differences in baseline motor speed (see lines 289-294).

13. **Methods - Participants] The manuscript reports an a priori power analysis (G*Power) suggesting N=20 for a repeated-measures ANOVA, yet the final sample is N=70 and the design includes a 2×2×2×4 within-subject structure. Please clarify which effect(s) the power analysis targeted (main effect vs. interaction), the assumed effect size, and key G*Power settings (e.g., correlation among repeated measures and nonsphericity correction). In addition, briefly explain the rationale for recruiting N=70 given the reported required sample size.**

- The a priori power analysis targeted the main effect of depiction type (realistic vs. line-drawing hands), which was the primary hypothesis of the study. The larger sample of 70 participants was recruited to ensure adequate power for detecting interaction effects, which typically require larger samples than main effects. Additionally, the 2 × 2 × 4 × 2 within-subjects design involves multiple conditions, and we anticipated the need for sufficient power to detect theoretically important higher-order interactions (e.g., View × Orientation × Depiction Type). The larger sample also provided a buffer against data loss due to exclusions based on high error rates (>30%) or technical issues.

14. **[Methods - Stimuli and Materials] While the description of the stimuli is generally clear, additional details would improve methodological transparency. Specifically, please clarify the number of trials per condition and whether trials were balanced across conditions. In addition, given that stimulus depiction type (photographs vs. line drawings) is a central manipulation of the study, reporting how stimulus properties such as luminance and contrast were controlled or matched across depiction types would strengthen the Methods section.**

- We have revised the Stimuli and Materials section to clarify the number of trials per condition and confirm that trials were balanced across all conditions

15. **[Methods - Procedure] The Procedure does not clearly specify the task instructions given during the HLJT experimental phase. In particular, it is unclear (i) whether participants were explicitly instructed to prioritize speed, accuracy, or a balance of both when making laterality judgments, and (ii) whether any guidance was provided regarding strategy use (e.g., imagining moving one’s own hand versus treating the stimulus as an object to be rotated). Given that reaction time and inferred strategy are central to the hypotheses and interpretation of the results, explicitly reporting these instructions would improve reproducibility and help readers evaluate how the observed RT patterns should be interpreted.**

- We have revised the Procedure section to explicitly state the task instructions provided during the experimental phase. We have revised the Procedure section to explicitly state that participants were instructed to respond, “as quickly and accurately as possible,” reflecting a balanced speed-accuracy emphasis. We have also clarified that no explicit guidance was provided regarding strategy use (see lines 241-245).

16. **[Methods - Data processing and analysis] The section titled “Data processing and analysis” does not describe any data preprocessing steps. Key aspects such as exclusion criteria for response times (e.g., incorrect trials, extreme RTs), handling of anticipatory or excessively slow responses, and any trial-level filtering are not specified. We suggest either (i) explicitly describing these preprocessing steps in this section, or (ii) revising the section title to more accurately reflect its content.**

- We have revised the Methods section to clearly separate data preprocessing from statistical analysis.

17. **[Methods - Independent Variables] The information provided in the ‘Independent Variables’ section substantially overlaps with the description in the Data Analysis section. The authors may consider integrating this information into a single, coherent description of the experimental design to avoid redundancy.**

- We have integrated the independent variables description into the Data Processing and Analysis section to avoid redundancy and provide a more coherent description of the experimental design.

18. **[Methods – Dependent measures] Reaction time and accuracy are both defined, but their respective theoretical roles are not clearly articulated. Given the emphasis on strategy differences (motor vs. visual imagery) throughout the manuscript, it would be helpful to clarify whether accuracy is treated as a secondary outcome, a complementary index of strategy use, or a critical test of the study’s hypotheses. Explicitly stating this would also help readers interpret the presence or absence of speed–accuracy trade-offs. As a robustness check, the authors may also consider reporting a sensitivity analysis that accounts for potential speed–accuracy trade-offs (e.g., a combined performance index such as inverse efficiency, or an alternative modeling approach that jointly considers RT and accuracy).**

- We have revised the Data Processing and Analysis section.

19. **[Figures 2-5] Figures 2–5 appear to present the core response time and accuracy patterns across view, orientation, and hand depiction type; however, they are not explicitly referenced or integrated into the Results text. The reported statistical effects are therefore not clearly linked to the corresponding figures. In addition, although the data are derived from a fully within-subject design, the figures are currently presented using condition-wise means and standard deviations only. While such summaries are acceptable, it would be helpful to additionally display individual participant values and connect corresponding data points across conditions to visually convey the paired nature of the data. Explicitly linking the Results text to the figures and revising the visualization to reflect within-subject structure would substantially improve clarity and interpretability.**

- We have revised the manuscript to explicitly reference Figures 2–5 within the Results text, ensuring that each reported statistical effect is clearly linked to its corresponding figure. Regarding the suggestion to display individual participant data points with connected lines, we opted to retain the current bar graph format for several reasons. First, with 62 participants and 32 conditions (2 laterality × 2 view × 4 orientation × 2 depiction type), displaying individual data points would result in visual clutter that obscures rather than clarifies the key patterns. Second, the use of SEM error bars appropriately conveys the within-subject precision of the estimates. Third, the primary theoretical interest of the study concerns condition-level effects (e.g., the medial-over-lateral advantage, view × depiction type interactions) rather than individual differences, making aggregate visualizations more appropriate for communicating the findings.

20. **[References] The ordering of references appears inconsistent with their first appearance in the manuscript. Please ensure that references are listed in the correct order according to the journal’s citation style and that in-text citations correspond appropriately to the reference list.**

- References have been updated.
