# Supplementary material for: The influence of hand depiction types on behavioural patterns in laterality judgments
Source: PLoS One. 2026 Jul 13;21(7):e0352694. doi: 10.1371/journal.pone.0352694 (PMC13362083; doi:10.1371/journal.pone.0352694)
Supplement: Attachment — Submitted filename: RevisionLetter_auresp_2.docx [file pone.0352694.s003.docx]

Dear Dr. Giulia Prete,

Thank you for the opportunity to revise our manuscript entitled “The Influence of Hand Depiction Types on Behavioural Patterns in Laterality Judgments”. We thank the reviewers for their thoughtful **comments and helpful suggestions.** Based on the suggested edits, we believe the manuscript has been significantly improved.

**In response to each comment, please find our responses below.** Within the edited document, we have referenced specific sections and page numbers where related edits (tracked changes) have been made.

**Reviewer 1:**

Thanks for considering all the review aspects provided. The changes are done rigorously without changing the paper in a not helpful way.

**Reviewer 2:**

1. **This study examines the effect of hand depiction type on performance in the HLJT; however, the theoretical foundation and rationale for the hypotheses remain insufficiently explained even in the revised manuscript. In particular, it is unclear what is meant by terms such as “most effective” (L.108) and “better suited” (L.196), that is, according to which metrics and criteria these evaluations are made. Therefore, substantial revision is still required. Additional specific concerns regarding the research objectives are outlined below.**

- Thank you for this comment. We revised the introduction and study aims to strengthen the theoretical rationale and clarify the objectives. In addition, wording suggesting that one stimulus type was ‘most effective’ or ‘better suited’ was removed and replaced with more precise language describing differences in behavioural performance

2. **In the Current Study section of the Introduction, heterogeneity of strategy use for back-view stimuli is emphasized (L.107); however, the present study is not designed to directly test this point, resulting in a mismatch between the introduction and the stated research objectives.**

- Thank you for this comment. We revised the current study and study Aims sections to better align the research objectives with the study design. Strategy heterogeneity is now presented as theoretical context rather than a directly tested hypothesis, and the study objectives were clarified accordingly.

3. **The rationale for the prediction that greater involvement of visual detail or biomechanical constraints would lead to longer response times is not convincingly explained.**

- We expanded the discussion of embodiment, ownership, visual detail, and biomechanical constraints and clarified how these factors may influence performance during hand laterality judgments instead of stating it would lead to longer response times.

4. **No clear justification is provided for the prediction that accuracy would not differ between conditions. If longer response times are interpreted as reflecting increased processing load, a decrease in accuracy would generally also be expected.**

- Thank you for this comment. We revised the Current Study section to avoid making an unsupported prediction that accuracy would not differ between conditions. Instead, the study objective and hypotheses are now framed more broadly around whether behavioural performance differs between realistic and line-drawing hand stimuli.

5. **The lack of clarity in the research objectives appears to stem from an insufficient organization of the existing literature, resulting in objectives that are not grounded in a well-established theoretical framework. In fact, there are several serious issues with the use and citation of prior studies, some examples of which are listed below. These issues go beyond minor errors or inconsistencies in reporting. Given that similar concerns were raised in the initial review, this is particularly concerning. The authors should recognize that proper organization of the literature is essential for establishing the positioning of the study and the validity of its hypotheses.**

- Thank you for this comment. We reorganized the Introduction and revised the Current Study section to provide a clearer theoretical framework and stronger connection between the literature review, study objectives, and hypotheses.

6. **In the description related to Parsons (L48), it is not specified which reference is being cited as the basis for the statement.**

- The manuscript has been revised to explicitly cite **Parsons (1987)** as the source supporting this statement.

7. **The citation numbering is inconsistent in terms of order of appearance and continuity, indicating that references have not been properly managed.**

- This has now been corrected.

8. **Some references listed (e.g., Ref.19 and Ref.21) may not actually be cited in the main text.**

- This has now been corrected.

9. **References cited in relation to left-handedness (Ref.20, 22) involve right-handed participants and do not support the statements made in the manuscript. In particular, Ref.22 may have been confused with another paper by the authors.**

- This has now been corrected.

10. **The paper by Mizuguchi N, et al. appears in the reference list (between Ref.18 and Ref.19) but is not assigned a citation number.**

- The reference by Mizuguchi et al. has now been assigned the appropriate citation number, and the numbering of the reference list has been corrected accordingly.

11. **It is unclear which claims are supported by which references, and the overall organization of citations is insufficient. For example, the references cited for line-drawn versus realistic hand stimuli (L.98) appear overly selective, as most prior studies using hand stimuli would fall into one of these categories. Similarly, the rationale behind the selection of references for explicit and implicit processes (L.99) is unclear.**

- This has now been corrected.

12. **Consistent with my initial review, there are fundamental issues with the statistical procedures in this study. In light of PLoS ONE’s emphasis on methodological rigor and the validity of interpretation, these issues are considered to be substantial.**

- Thank you for this comment. We revised the analytic approach to examine palm- and back-view stimuli separately, consistent with theoretical distinctions between these stimulus types. The Methods, Results, and interpretation of findings were revised accordingly.

13. **In response to the initial review, the authors state that, in addition to a power analysis targeting main effects, the sample size was increased to account for the detection of interactions. However, this explanation lacks quantitative justification, and it remains unclear whether the current sample size is adequate for the multifactorial analyses actually conducted in this study, including higher-order interactions. In other words, the relationship between the a priori design (power analysis) and the actual analytical strategy remains unclear. The authors should clarify the target effects and underlying assumptions of the power analysis, or alternatively, indicate the range of effect sizes detectable with the current sample size in order to reconstruct the validity of the study design.**

- The original a priori power analysis was conducted to detect a medium-to-large repeated-measures effect (f = .25, α = .05, power = .80), yielding a minimum sample size of 20 participants. The final sample of 62 participants substantially exceeded this requirement. In addition, the revised analyses examined palm- and back-view stimuli separately, reducing model complexity relative to the original multifactorial approach.

14. **This study employs a multifactorial analysis including a four-way interaction; however, when a higher-order interaction is significant, main effects cannot be interpreted as general tendencies, and the effects of each factor should instead be interpreted as conditional. In the present manuscript, main effect–like descriptions are provided regardless of the presence of interactions, indicating that the principle of hierarchical interpretation is not adequately followed. The authors should clearly distinguish the level at which interpretation is appropriate based on the results of higher-order interactions and restructure the presentation of the results accordingly. In particular, for response time, the manuscript states that when higher-order interactions are significant, the data are decomposed by view (palm vs. back). However, the subsequent analyses and reporting are not consistent with this stated approach. If the data are indeed decomposed by view, analyses should proceed separately within Palm and Back conditions using the remaining factors (laterality, orientation, stimulus type), effectively as three-way ANOVAs, in which case view should no longer appear as an analysis factor. In contrast, interactions involving view continue to be reported, indicating that the analytical procedure is not applied consistently. This inconsistency suggests that the principle of hierarchical interpretation of interactions is not properly maintained.**

- Thank you for this comment. In the revised manuscript, palm- and back-view stimuli are analyzed separately, consistent with the decomposition approach described in the Methods. Accordingly, View is no longer included as a factor in the reported analyses, and all response time results are presented and interpreted within separate Palm View and Back View analyses. The Results section was reorganized to ensure consistency between the analytic approach and interpretation of interaction effects.

15. **Furthermore, although the study assumes that different processing mechanisms underlie palm and back views, it remains unclear how these two conditions are compared and positioned within the analysis. If a direct comparison between palm and back views is not the primary objective, it may be more appropriate to separate the data by view in advance and evaluate the effects of the remaining factors (laterality, orientation, stimulus type) within each view. Indeed, prior studies have adopted such an approach. Therefore, the authors should consider restructuring the analysis as a three-way ANOVA (Laterality × Orientation × Stimulus Type) within each view and interpreting the results accordingly. Clarifying the relationship between the analytical strategy and the research objectives in this manner would help ensure consistency in the interpretation of the findings.**

- Thank you for this comment. We revised the analytic approach to examine palm- and back-view stimuli separately using view-specific analyses. Accordingly, response time data are now analyzed within each view using factors of Laterality, Orientation, and Stimulus Type, and View is no longer treated as a factor in the reported analyses. The Results section and interpretation of findings were reorganized accordingly to ensure consistency between the analytical strategy and research objectives.

**Minor Comments**

16. **[Abstract] The Abstract is generally clear; however, the results are described in terms of main effects, which may not be consistent with the interaction structure reported in the main text. In particular, the effects of depiction type and view are presented as general tendencies, although they are likely to be condition-dependent. Therefore, the Abstract should be revised to align with the statistical structure of the results**.

- The Abstract has been revised to better reflect the interaction structure of the results. Specifically, we now emphasize that the effects of hand depiction type varied as a function of viewpoint and orientation and report the condition-specific findings separately for back-view and palm-view stimuli.

17. **[Introduction] The extrastriate body area (EBA) is introduced; however, it is not sufficiently integrated into the study’s hypotheses or the interpretation of the results. Its role should be clarified or the description should be reorganized.**

- We thank the reviewer for this comment. The discussion of the extrastriate body area (EBA) was included to provide background regarding neural systems implicated in body-part perception and hand mental rotation.

18. **[Participants] The relationship between the number of recruited participants (62) and those who received experimental credit (70) is unclear and may be inconsistent (L.131). It should be clarified whether these refer to the same sample or different populations.**

- We thank the reviewer for this comment. The Participants section has been revised to clarify the distinction between the recruited sample (N = 70) and the final analyzed sample (N = 62)

19. **[Stimuli and Materials] It is recommended to provide additional details regarding display resolution and stimulus size. Although differences in visual characteristics between depiction types are mentioned in the Discussion, the information provided is insufficient. These details are also important for reproducibility.**

- Details regarding the visual characteristics of the realistic and line-drawing stimuli have been added to the Stimuli and Materials section to improve reproducibility and clarify differences between the stimulus types.

20. **[Procedure] Although not pointed out in the initial review, the inclusion of a task in training phase 1 that requires participants to physically move their hands to match the stimuli may have induced motor imagery during the main task. As this could affect the interpretation of the results, it is recommended that this possibility be discussed in the Discussion section.**

- A brief discussion of this issue has been added to the Discussion section.

21. **[Data processing and analysis – Data exclusion] It is recommended to clarify the rationale for the response time exclusion criteria, either by citing relevant prior studies or by providing a brief justification.**

- A brief justification for the response time exclusion criteria has been added to the Data Exclusion section.

22. **[Results - Simple reaction time (SRT)] As this is the Results section, the description should be limited to the presentation of results. The statement that SRT values were not used in subsequent analyses is methodological in nature and should be described in the Methods if necessary. Furthermore, in the absence of statistical testing, the description remains impressionistic and is not appropriate for this section. Any interpretative or suggestive content should instead be addressed in the Discussion.**

- The SRT section has been revised to report only descriptive SRT values, and methodological information has been removed from the Results section.

23. **[Figures] The role of the figures in relation to the statistical analyses is unclear. Given that higher-order interactions are reported, figures should be structured to reflect condition-dependent effects rather than general trends. However, the current presentation may give the impression of main effects or overall tendencies, which is not consistent with the reported interaction structure. The authors should clarify the purpose of each figure and ensure that the graphical presentation is aligned with the statistical model and its hierarchical interpretation.**

- The Results section was reorganized to present palm- and back-view analyses separately, and the figures are now presented in a manner that reflects the condition-specific effects reported in the statistical analyses.
