# Supplementary material for: The influence of hand depiction types on behavioural patterns in laterality judgments
Source: PLoS One. 2026 Jul 13;21(7):e0352694. doi: 10.1371/journal.pone.0352694 (PMC13362083; doi:10.1371/journal.pone.0352694)
Supplement: Attachment — Submitted filename: RevisionLetter_auresp_3.docx [file pone.0352694.s004.docx]

Dear Dr. Giulia Prete,

Thank you for the opportunity to revise our manuscript entitled “The Influence of Hand Depiction Types on Behavioural Patterns in Laterality Judgments”. We thank the reviewers for their thoughtful **comments and helpful suggestions.** Based on the suggested edits, we believe the manuscript has been significantly improved.

**In response to each comment, please find our responses below.** Within the edited document, we have referenced specific sections and page numbers where related edits (tracked changes) have been made.

**Editor: As you can seen, I re-invited the expert who revised the previous version of the manuscript. He/She acknowledges that the manuscript is now improved but also highlighted serious issues. Since the other reviewer had recommended acceptance of the study, I decided not to reject it at this stage. However, I would like to strongly suggest you to fully consider all the present suggestions. Please, pay attention to citations in particular (see Reviewer's comment), as well as to hierarchical interpretation of interaction effects. I will evaluate the next revisions myself before deciding how to proceed.**

- We would also like to note that the current manuscript underwent substantial revision in response to Reviewer 2’s previous comments. Specifically, the data were reanalyzed using a revised analytical approach, including a reduction from a four-factor to a three-factor framework, and the Results and Discussion sections were extensively rewritten to reflect these changes. We are grateful for the reviewer’s continued feedback and have carefully considered the current comments to further improve the manuscript.

**Reviewer 2:**

1. **The overall organization of the manuscript has improved substantially since the previous revision, and the authors' efforts are appreciated. However, a number of serious problems with the citation of prior literature have been identified. Most critically, one of the references cited in the manuscript (Reference [30]) could not be verified by title, author names, DOI, or direct inspection of the relevant issue of the journal. The citation of a publication that cannot be confirmed to exist raises serious concerns regarding scientific integrity, and makes it difficult to evaluate this manuscript as a scientific paper. Furthermore, numerous instances were found in which the content of cited works does not align with the context in which they are cited. This issue was raised in the previous round of review, and the fact that it remains unresolved after revision suggests a fundamental insufficiency in the authors' understanding and organization of the relevant literature.**
   - We thank the reviewer for identifying this issue. The Ferri et al. paper does exist; however, an error in the reference information during manuscript revision made the citation difficult to verify.
   - We carefully reviewed all citations throughout the manuscript and revised several statements to ensure that the cited literature more accurately reflected the claims being made. We also corrected the reference-list error involving the Ferri et al. citation.
2. **The rationale for the research objectives also remains insufficiently developed. It is unclear why this study is necessary and how the proposed objectives follow logically from the existing literature, resulting in a disconnect between the Introduction and the Methods. In addition, simple reaction time (SRT) is measured and reported in the manuscript, yet its purpose is not stated in the Methods, and no interpretation is offered in the Discussion. The failure to account for the significance of a measured variable within the manuscript raises concerns regarding the coherence of the study design.**

- To address this concern, we strengthened the rationale for the study by more clearly identifying the gap in the literature and explaining the importance of examining hand depiction type in the HLJT. Specifically, we revised the current study section (Lines 109–113) to better link the reviewed literature, the identified gap, and the objectives of the present study.
- With respect to simple reaction time (SRT), we have clarified its purpose in the Methods section (Lines 184–186). We also added text to the Discussion (Lines 295–297) noting that the relatively small differences observed in SRT suggest that the reported HLJT effects are unlikely to reflect general differences in baseline motor speed.

3. **The statistical procedures have been improved in the revised manuscript. However, the problem of interpreting findings as general tendencies without adequately accounting for the conditional nature of effects indicated by significant interactions remains unresolved, and the hierarchical interpretation of interaction effects cannot be considered appropriate.**

- In the previously revised manuscript, palm- and back-view stimuli were analyzed separately, and significant interactions were followed up using Bonferroni-corrected pairwise comparisons. The Results and Discussion sections were structured around these decomposed analyses, and findings were interpreted within the context of the relevant view-, orientation-, and laterality-specific effects rather than as general tendencies. We have carefully reviewed the manuscript and believe that the current interpretation appropriately reflects the conditional nature of the observed effects.
