# Supplementary material for: The influence of hand depiction types on behavioural patterns in laterality judgments
Source: PLoS One. 2026 Jul 13;21(7):e0352694. doi: 10.1371/journal.pone.0352694 (PMC13362083; doi:10.1371/journal.pone.0352694)
Supplement: Peer review history

# Peer review: Decision Letter 0

**DOI:** 10.1371/journal.pone.0352694.r001

**Authors:** Giulia Prete

27 Jan 2026

PONE-D-25-62785

The Influence of Hand Depiction Types on Behavioural Patterns in Laterality Judgments

PLOS One

Dear Dr. Saran,

Thank you for submitting your manuscript to PLOS ONE. After careful consideration, we feel that it has merit but does not fully meet PLOS ONE’s publication criteria as it currently stands. Therefore, we invite you to submit a revised version of the manuscript that addresses the points raised during the review process.

As you can see below, two expert reviewers have evaluated your study. They consider the experimental idea interesting, but recommend substantial revision to improve the manuscript’s clarity. I would like to thank the reviewers for their very constructive comments and urge you to seriously consider their suggestions, including more detail on data preprocessing and analytical decision rules, the hierarchical logic needed to interpret higher-order interactions, and the rewording of the introduction and discussion to be more logical and concise.

Please submit your revised manuscript by Mar 13 2026 11:59PM. If you will need more time than this to complete your revisions, please reply to this message or contact the journal office at plosone@plos.org. When you're ready to submit your revision, log on to https://www.editorialmanager.com/pone/ and select the 'Submissions Needing Revision' folder to locate your manuscript file.

Please include the following items when submitting your revised manuscript:

- A letter that responds to each point raised by the academic editor and reviewer(s). You should upload this letter as a separate file labeled 'Response to Reviewers'.
- A marked-up copy of your manuscript that highlights changes made to the original version. You should upload this as a separate file labeled 'Revised Manuscript with Track Changes'.
- An unmarked version of your revised paper without tracked changes. You should upload this as a separate file labeled 'Manuscript'.

If you would like to make changes to your financial disclosure, please include your updated statement in your cover letter. Guidelines for resubmitting your figure files are available below the reviewer comments at the end of this letter.

If applicable, we recommend that you deposit your laboratory protocols in protocols.io to enhance the reproducibility of your results. Protocols.io assigns your protocol its own identifier (DOI) so that it can be cited independently in the future. For instructions see: https://journals.plos.org/plosone/s/submission-guidelines#loc-laboratory-protocols. Additionally, PLOS ONE offers an option for publishing peer-reviewed Lab Protocol articles, which describe protocols hosted on protocols.io. Read more information on sharing protocols at https://plos.org/protocols?utm_medium=editorial-email&utm_source=authorletters&utm_campaign=protocols.

We look forward to receiving your revised manuscript.

Kind regards,

Giulia Prete

Academic Editor

PLOS One

Journal Requirements:

When submitting your revision, we need you to address these additional requirements.

1. Please ensure that your manuscript meets PLOS ONE's style requirements, including those for file naming. The PLOS ONE style templates can be found at

https://journals.plos.org/plosone/s/file?id=wjVg/PLOSOne_formatting_sample_main_body.pdf and

https://journals.plos.org/plosone/s/file?id=ba62/PLOSOne_formatting_sample_title_authors_affiliations.pdf

2. Please note that PLOS One has specific guidelines on code sharing for submissions in which author-generated code underpins the findings in the manuscript. In these cases, we expect all author-generated code to be made available without restrictions upon publication of the work. Please review our guidelines at https://journals.plos.org/plosone/s/materials-and-software-sharing#loc-sharing-code and ensure that your code is shared in a way that follows best practice and facilitates reproducibility and reuse.

3. Please include captions for your Supporting Information files at the end of your manuscript, and update any in-text citations to match accordingly. Please see our Supporting Information guidelines for more information: http://journals.plos.org/plosone/s/supporting-information.

4. If the reviewer comments include a recommendation to cite specific previously published works, please review and evaluate these publications to determine whether they are relevant and should be cited. There is no requirement to cite these works unless the editor has indicated otherwise.

[Note: HTML markup is below. Please do not edit.]

Reviewers' comments:

Reviewer's Responses to Questions

**Comments to the Author**

1. Is the manuscript technically sound, and do the data support the conclusions?

The manuscript must describe a technically sound piece of scientific research with data that supports the conclusions. Experiments must have been conducted rigorously, with appropriate controls, replication, and sample sizes. The conclusions must be drawn appropriately based on the data presented.

Reviewer #1: Yes

Reviewer #2: No

**********

2. Has the statistical analysis been performed appropriately and rigorously?

Reviewer #1: Yes

Reviewer #2: No

**********

3. Have the authors made all data underlying the findings in their manuscript fully available?

The PLOS Data policy requires authors to make all data underlying the findings described in their manuscript fully available without restriction, with rare exception (please refer to the Data Availability Statement in the manuscript PDF file). The data should be provided as part of the manuscript or its supporting information, or deposited to a public repository. For example, in addition to summary statistics, the data points behind means, medians and variance measures should be available. If there are restrictions on publicly sharing data—e.g. participant privacy or use of data from a third party—those must be specified.

Reviewer #1: Yes

Reviewer #2: Yes

**********

4. Is the manuscript presented in an intelligible fashion and written in standard English?

PLOS ONE does not copyedit accepted manuscripts, so the language in submitted articles must be clear, correct, and unambiguous. Any typographical or grammatical errors should be corrected at revision, so please note any specific errors here.

Reviewer #1: Yes

Reviewer #2: Yes

**********

5. Review Comments to the Author

Please use the space provided to explain your answers to the questions above. You may also include additional comments for the author, including concerns about dual publication, research ethics, or publication ethics. (Please upload your review as an attachment if it exceeds 20,000 characters)

Reviewer #1: PLOS One

The Influence of Hand Depiction Types on Behavioural Patterns in Laterality Judgements

PONE-D-25-62785

Abstract:

The aim of the study should be stated on the described background. Also a brief discussion of the results and of its scientific and clinical worth should be provided.

Introduction:

49-78: This paragraph can and should be shortened to the most important information required to understand the aim of the study.

79-150: Though the provided information is very interesting it is not clear how it supports the study and the rest of the article, especially regarding the detailed explanations. Please shorten this paragraph as well in a clear and supportive way.

151-192: Here the proplem definition is provided and it should be stated such as. Skip the term hypothesis as it is not a thesis but a scientific article. Provide the aim of the study with 3 different aims in a more compact and shortened way.

Methods:

194-198: Provide the information to statistics at the end of the method section

214-216: No results in the method section!

199-219: Please provide exclusion criteria

260-291: Shorten this paragraph, it is not a thesis. See comment above to 194-198

Results:

Please be clear with the term “more errors” – this is not very accurate.

Discussion:

There is quite an extended interpretation of the results without further ideas regarding scientific purpose or clinical purpose. Please try to find the following structure for your discussion:

- Key findings

- Your results in the context of previous works

- Limitations

- Strengths

- Ways to move the field further

Summary:

Try to be more consistent and keep the information to a relevant amount of content. Though many of this information is interesting to you, be aware of the reader, and how much does it need to understand your aim of the study and the interpretation of your results. It might be interesting if the authors have any clinical ideas (see discussion point ways to move the field further) – so far the relevance of their results remain sparse, especially beyond the field of testing laterality.

Reviewer #2: General Comments

This manuscript addresses an interesting question regarding the role of hand depiction type in the hand laterality judgment task. However, I have serious concerns about the transparency and structure of the Methods and Results sections. Essential details regarding data preprocessing and analytical decision rules are missing, and the hierarchical logic required to interpret effects in a four-factor design is not clearly respected. In particular, the outcome of the highest-order (four-way) interaction is not reported, which makes it difficult to evaluate the validity of the reported lower-order effects.

Because these methodological and analytical issues substantially limit the interpretability of the results, I have not commented in detail on the Discussion section. I believe that the Methods and Results require substantial revision before the theoretical implications of the findings can be meaningfully assessed.

Major Comments

1� [Introduction - Kinesthetic Motor vs. Visual Motor Strategy] The dichotomous classification of imagery strategies into kinesthetic (first-person) for palm views and visual (third-person) for back views appears overly simplified. Previous studies have suggested that responses to back-view stimuli may rely not only on visual motor imagery, but also on pattern matching or object-like mental rotation processes, similar to those used for non-body objects. While I agree that kinesthetic motor imagery is likely dominant for palm-view stimuli, the strategy employed for back-view stimuli may be more heterogeneous than described here. We therefore suggest that this section be substantially revised to reflect a more nuanced view of imagery strategies, and to more carefully integrate relevant prior literature.

2� [Introduction - The Current Study] While the Introduction provides a comprehensive overview of biomechanical constraints and imagery strategies in the HLJT, the rationale for focusing on hand depiction type is not sufficiently developed. Given the strong emphasis on embodied and motor-based processing throughout the Introduction, it would strengthen the manuscript to more explicitly explain why differences in stimulus realism are theoretically relevant, and how hand depiction type relates to the previously discussed mechanisms. Clarifying this link would make the motivation for the current study more compelling.

3� [Introduction - Hypotheses] Hypotheses 1 and 2 largely restate well-established phenomena in the HLJT literature, including canonical orientation effects and the medial-over-lateral advantage (MOLA), rather than articulating hypotheses that advance or differentiate the aims of the present study. As such, these statements read more like background assumptions than hypotheses and should be reconsidered accordingly. Hypothesis 3 is closer to a study-specific prediction than Hypotheses 1 and 2; however, it currently conflates hypotheses with explanatory statements and general assumptions about motor rules. The predicted direction of the reaction time effect (slower responses for realistic hands) is not sufficiently justified, the rationale for preserved accuracy remains unclear, and view-dependent differences emphasized earlier in the Introduction are not adequately accounted for. The authors should substantially refine Hypothesis 3 to focus on a clear, falsifiable prediction directly tied to hand depiction type.

4� [Methods - Data analysis] The description of data handling and statistical analysis lacks essential methodological detail. First, the manuscript does not specify how incorrect responses and extreme RT outliers were treated, despite these procedures being critical for RT-based analyses. Second, reaction time and accuracy were analyzed using different statistical frameworks (repeated-measures ANOVA vs. GLMM), but the rationale for this discrepancy is not explained. Third, the analytical strategy for handling interactions is insufficiently specified; it remains unclear how the presence or absence of interactions guided subsequent analyses and interpretation (e.g., whether and how simple effects were examined). Explicitly defining these procedures is necessary to ensure transparency, reproducibility, and interpretability of the results.

5� [Results – Response times] Reaction time and accuracy were analyzed using multi-factor models including Laterality, View, Orientation, and Hand Type. In such a four-factor design, I believe that the interpretation of effects should follow a clear hierarchical structure, beginning with the highest-order interaction. Specifically, the presence or absence of a four-way interaction (Laterality × View × Orientation × Hand Type) determines whether lower-order interactions and main effects can be meaningfully interpreted as general effects or only as conditional effects.

In the current Results sections for both RT and accuracy, the outcome of the four-way interaction is not reported. Instead, selected three-way and two-way interactions are described, followed by unqualified main effects. Without explicitly stating whether the four-way interaction was tested and whether it was significant, I find it difficult to evaluate the scope and validity of the reported lower-order effects.

Moreover, multiple three-way interactions are possible in this design, yet only a subset is reported, and the criteria for selecting and decomposing these interactions are not clearly specified. When higher-order interactions are significant, it is standard practice to either (i) focus interpretation on those higher-order effects and their planned decompositions (e.g., via simple-effects analyses), or (ii) explicitly justify why certain lower-order effects are examined independently.

I therefore recommend that the authors (1) explicitly report the results of the four-way interaction for both RT and accuracy, (2) clarify a priori decision rules for proceeding from four-way to three-way and lower-order analyses, and (3) restructure the Results sections to reflect this hierarchical logic. Doing so would substantially improve the transparency, interpretability, and reproducibility of the findings.

Minor Comments

6� [Abstract] The abstract is necessarily concise and generally reflects the main findings; however, it somewhat underrepresents the hypothesis-driven nature of the study and the role of accuracy analyses.

7� [Abstract] While the abstract is necessarily concise, the use of abbreviations such as “90°L” may not be self-explanatory to a broad readership. A brief clarification (e.g., lateral orientation) would improve the clarity of the abstract.

8� [Introduction - 1st paragraph] In the introductory description of the HLJT, the authors cite a preprint reference (Ref. 14). Given that this passage serves as a general definition of the task, it may be more appropriate to cite a canonical or review paper on the HLJT.

9� [Introduction - 1st paragraph] The explanatory section from the push-up example through the introduction of MOLA would benefit from substantial clarification. In its current form, the discussion mixes geometric notions of rotation with biomechanical constraints, which makes the underlying rationale difficult to follow. Reorganizing this section to emphasize biomechanical asymmetries as the core explanation for MOLA would improve conceptual clarity.

10� [Introduction - 1st paragraph] Ref. 15 may concern a foot laterality task; its use to support arm/shoulder pain may require clarification.

11� [Methods - 1st paragraph] The opening paragraph of the Methods section describes the experimental design using an ANOVA framework. While the statistical analysis itself is explained later, presenting the design primarily in terms of the analysis may blur the distinction between experimental design and statistical procedures. Clarifying the design independently of the ANOVA terminology (e.g., explicitly framing it as a factorial design) would improve conceptual clarity for the reader.

12� [Methods - Participants] The simple reaction time (SRT) is reported, but it does not appear to be incorporated into subsequent analyses. Some prior HLJT studies adjust for individual differences in basic motor speed (e.g., by subtracting or otherwise controlling for simple RT). Please clarify the intended role of the SRT measure in the present study (descriptive only vs. used for adjustment), and, if not used, briefly justify why (and/or consider reporting analyses with motor-speed adjustment as a robustness check).

13� [Methods - Participants] The manuscript reports an a priori power analysis (G*Power) suggesting N=20 for a repeated-measures ANOVA, yet the final sample is N=70 and the design includes a 2×2×2×4 within-subject structure. Please clarify which effect(s) the power analysis targeted (main effect vs. interaction), the assumed effect size, and key G*Power settings (e.g., correlation among repeated measures and nonsphericity correction). In addition, briefly explain the rationale for recruiting N=70 given the reported required sample size.

14� [Methods - Stimuli and Materials] While the description of the stimuli is generally clear, additional details would improve methodological transparency. Specifically, please clarify the number of trials per condition and whether trials were balanced across conditions. In addition, given that stimulus depiction type (photographs vs. line drawings) is a central manipulation of the study, reporting how stimulus properties such as luminance and contrast were controlled or matched across depiction types would strengthen the Methods section.

15� [Methods - Procedure] The Procedure does not clearly specify the task instructions given during the HLJT experimental phase. In particular, it is unclear (i) whether participants were explicitly instructed to prioritize speed, accuracy, or a balance of both when making laterality judgments, and (ii) whether any guidance was provided regarding strategy use (e.g., imagining moving one’s own hand versus treating the stimulus as an object to be rotated). Given that reaction time and inferred strategy are central to the hypotheses and interpretation of the results, explicitly reporting these instructions would improve reproducibility and help readers evaluate how the observed RT patterns should be interpreted.

16� [Methods - Data processing and analysis] The section titled “Data processing and analysis” does not describe any data preprocessing steps. Key aspects such as exclusion criteria for response times (e.g., incorrect trials, extreme RTs), handling of anticipatory or excessively slow responses, and any trial-level filtering are not specified. We suggest either (i) explicitly describing these preprocessing steps in this section, or (ii) revising the section title to more accurately reflect its content.

17� [Methods - Independent Variables] The information provided in the ‘Independent Variables’ section substantially overlaps with the description in the Data Analysis section. The authors may consider integrating this information into a single, coherent description of the experimental design to avoid redundancy.

18� [Methods – Dependent measures] Reaction time and accuracy are both defined, but their respective theoretical roles are not clearly articulated. Given the emphasis on strategy differences (motor vs. visual imagery) throughout the manuscript, it would be helpful to clarify whether accuracy is treated as a secondary outcome, a complementary index of strategy use, or a critical test of the study’s hypotheses. Explicitly stating this would also help readers interpret the presence or absence of speed–accuracy trade-offs. As a robustness check, the authors may also consider reporting a sensitivity analysis that accounts for potential speed–accuracy trade-offs (e.g., a combined performance index such as inverse efficiency, or an alternative modeling approach that jointly considers RT and accuracy).

19� [Figures 2-5] Figures 2–5 appear to present the core response time and accuracy patterns across view, orientation, and hand depiction type; however, they are not explicitly referenced or integrated into the Results text. The reported statistical effects are therefore not clearly linked to the corresponding figures. In addition, although the data are derived from a fully within-subject design, the figures are currently presented using condition-wise means and standard deviations only. While such summaries are acceptable, it would be helpful to additionally display individual participant values and connect corresponding data points across conditions to visually convey the paired nature of the data. Explicitly linking the Results text to the figures and revising the visualization to reflect within-subject structure would substantially improve clarity and interpretability.

20� [References] The ordering of references appears inconsistent with their first appearance in the manuscript. Please ensure that references are listed in the correct order according to the journal’s citation style and that in-text citations correspond appropriately to the reference list.

**********

6. PLOS authors have the option to publish the peer review history of their article (what does this mean?). If published, this will include your full peer review and any attached files.

If you choose “no”, your identity will remain anonymous but your review may still be made public.

**Do you want your identity to be public for this peer review?**  For information about this choice, including consent withdrawal, please see our Privacy Policy.

Reviewer #1: No

Reviewer #2: No

**********

[NOTE: If reviewer comments were submitted as an attachment file, they will be attached to this email and accessible via the submission site. Please log into your account, locate the manuscript record, and check for the action link "View Attachments". If this link does not appear, there are no attachment files.]

To ensure your figures meet our technical requirements, please review our figure guidelines: https://journals.plos.org/plosone/s/figures

You may also use PLOS’s free figure tool, NAAS, to help you prepare publication quality figures: https://journals.plos.org/plosone/s/figures#loc-tools-for-figure-preparation.

NAAS will assess whether your figures meet our technical requirements by comparing each figure against our figure specifications.

# Author comment: Author response to Decision Letter 1

**DOI:** 10.1371/journal.pone.0352694.r002

9 Mar 2026

Please see the attached revision letter that responds to specific reviewer and editor comments.

Attachment Submitted filename: RevisionLetter.docx

# Peer review: Decision Letter 1

**DOI:** 10.1371/journal.pone.0352694.r003

**Authors:** Giulia Prete, Giulia Prete

30 Mar 2026

PONE-D-25-62785R1

The Influence of Hand Depiction Types on Behavioural Patterns in Laterality Judgments

PLOS One

Dear Dr. Saran,

Thank you for submitting your manuscript to PLOS ONE. After careful consideration, we feel that it has merit but does not fully meet PLOS ONE’s publication criteria as it currently stands. Therefore, we invite you to submit a revised version of the manuscript that addresses the points raised during the review process.

Please submit your revised manuscript by May 14 2026 11:59PM. If you will need more time than this to complete your revisions, please reply to this message or contact the journal office at plosone@plos.org. When you're ready to submit your revision, log on to https://www.editorialmanager.com/pone/ and select the 'Submissions Needing Revision' folder to locate your manuscript file.

Please include the following items when submitting your revised manuscript:

- A letter that responds to each point raised by the academic editor and reviewer(s). You should upload this letter as a separate file labeled 'Response to Reviewers'.
- A marked-up copy of your manuscript that highlights changes made to the original version. You should upload this as a separate file labeled 'Revised Manuscript with Track Changes'.
- An unmarked version of your revised paper without tracked changes. You should upload this as a separate file labeled 'Manuscript'.

If you would like to make changes to your financial disclosure, please include your updated statement in your cover letter. Guidelines for resubmitting your figure files are available below the reviewer comments at the end of this letter.

If applicable, we recommend that you deposit your laboratory protocols in protocols.io to enhance the reproducibility of your results. Protocols.io assigns your protocol its own identifier (DOI) so that it can be cited independently in the future. For instructions see: https://journals.plos.org/plosone/s/submission-guidelines#loc-laboratory-protocols. Additionally, PLOS ONE offers an option for publishing peer-reviewed Lab Protocol articles, which describe protocols hosted on protocols.io. Read more information on sharing protocols at https://plos.org/protocols?utm_medium=editorial-email&utm_source=authorletters&utm_campaign=protocols.

As the corresponding author, your ORCID iD is verified in the submission system and will appear in the published article. PLOS supports the use of ORCID, and we encourage all coauthors to register for an ORCID iD and use it as well. Please encourage your coauthors to verify their ORCID iD within the submission system before final acceptance, as unverified ORCID iDs will not appear in the published article. *Only*  the individual author can complete the verification step; PLOS staff *cannot*  verify ORCID iDs on behalf of authors.

We look forward to receiving your revised manuscript.

Kind regards,

Giulia Prete

Academic Editor

PLOS One

**Journal Requirements:**

If the reviewer comments include a recommendation to cite specific previously published works, please review and evaluate these publications to determine whether they are relevant and should be cited. There is no requirement to cite these works unless the editor has indicated otherwise.

[Note: HTML markup is below. Please do not edit.]

Reviewers' comments:

Reviewer's Responses to Questions

**Comments to the Author**

1. If the authors have adequately addressed your comments raised in a previous round of review and you feel that this manuscript is now acceptable for publication, you may indicate that here to bypass the “Comments to the Author” section, enter your conflict of interest statement in the “Confidential to Editor” section, and submit your "Accept" recommendation.

Reviewer #1: All comments have been addressed

Reviewer #2: (No Response)

**********

2. Is the manuscript technically sound, and do the data support the conclusions?

The manuscript must describe a technically sound piece of scientific research with data that supports the conclusions. Experiments must have been conducted rigorously, with appropriate controls, replication, and sample sizes. The conclusions must be drawn appropriately based on the data presented.

Reviewer #1: Yes

Reviewer #2: No

**********

3. Has the statistical analysis been performed appropriately and rigorously?

Reviewer #1: Yes

Reviewer #2: No

**********

4. Have the authors made all data underlying the findings in their manuscript fully available?

The PLOS Data policy requires authors to make all data underlying the findings described in their manuscript fully available without restriction, with rare exception (please refer to the Data Availability Statement in the manuscript PDF file). The data should be provided as part of the manuscript or its supporting information, or deposited to a public repository. For example, in addition to summary statistics, the data points behind means, medians and variance measures should be available. If there are restrictions on publicly sharing data—e.g. participant privacy or use of data from a third party—those must be specified.

Reviewer #1: Yes

Reviewer #2: (No Response)

**********

5. Is the manuscript presented in an intelligible fashion and written in standard English?

PLOS ONE does not copyedit accepted manuscripts, so the language in submitted articles must be clear, correct, and unambiguous. Any typographical or grammatical errors should be corrected at revision, so please note any specific errors here.

Reviewer #1: Yes

Reviewer #2: Yes

**********

6. Review Comments to the Author

Please use the space provided to explain your answers to the questions above. You may also include additional comments for the author, including concerns about dual publication, research ethics, or publication ethics. (Please upload your review as an attachment if it exceeds 20,000 characters)

**Reviewer #1:** Thanks for considering all the review aspects provided.

The changes are done rigorously without changing the paper in a not helpful way.

**Reviewer #2:**  General Comments

This study addresses an interesting question regarding the effect of hand depiction type on performance in the hand laterality judgment task. However, even in the revised manuscript, the theoretical basis of the research objectives and hypotheses remains insufficiently developed, and the connection to previous studies is unclear. In addition, the handling of higher-order interactions in the statistical analyses is not consistent with the interpretation of the results, resulting in a lack of coherence between the analytical strategy and the reporting of findings. This issue directly affects the interpretability of the study, making it difficult to adequately evaluate the validity of the conclusions in its current form. Furthermore, because revisions to the methods and analyses may alter the results themselves, I have not provided detailed comments on the Discussion. Overall, substantial revision is required, including clarification of the research objectives as well as reorganization of the statistical analyses and the presentation of results.

Major Comments

1. This study examines the effect of hand depiction type on performance in the HLJT; however, the theoretical foundation and rationale for the hypotheses remain insufficiently explained even in the revised manuscript. In particular, it is unclear what is meant by terms such as “most effective” (L.108) and “better suited” (L.196), that is, according to which metrics and criteria these evaluations are made. Therefore, substantial revision is still required. Additional specific concerns regarding the research objectives are outlined below.

- In the Current Study section of the Introduction, heterogeneity of strategy use for back-view stimuli is emphasized (L.107); however, the present study is not designed to directly test this point, resulting in a mismatch between the introduction and the stated research objectives.

- The rationale for the prediction that greater involvement of visual detail or biomechanical constraints would lead to longer response times is not convincingly explained.

- No clear justification is provided for the prediction that accuracy would not differ between conditions. If longer response times are interpreted as reflecting increased processing load, a decrease in accuracy would generally also be expected.

The lack of clarity in the research objectives appears to stem from an insufficient organization of the existing literature, resulting in objectives that are not grounded in a well-established theoretical framework. In fact, there are several serious issues with the use and citation of prior studies, some examples of which are listed below. These issues go beyond minor errors or inconsistencies in reporting. Given that similar concerns were raised in the initial review, this is particularly concerning. The authors should recognize that proper organization of the literature is essential for establishing the positioning of the study and the validity of its hypotheses.

- In the description related to Parsons (L48), it is not specified which reference is being cited as the basis for the statement.

- The citation numbering is inconsistent in terms of order of appearance and continuity, indicating that references have not been properly managed.

- Some references listed (e.g., Ref.19 and Ref.21) may not actually be cited in the main text.

- References cited in relation to left-handedness (Ref.20, 22) involve right-handed participants and do not support the statements made in the manuscript. In particular, Ref.22 may have been confused with another paper by the authors.

- The paper by Mizuguchi N, et al. appears in the reference list (between Ref.18 and Ref.19) but is not assigned a citation number.

- It is unclear which claims are supported by which references, and the overall organization of citations is insufficient. For example, the references cited for line-drawn versus realistic hand stimuli (L.98) appear overly selective, as most prior studies using hand stimuli would fall into one of these categories. Similarly, the rationale behind the selection of references for explicit and implicit processes (L.99) is unclear.

2. Consistent with my initial review, there are fundamental issues with the statistical procedures in this study. In light of PLoS ONE’s emphasis on methodological rigor and the validity of interpretation, these issues are considered to be substantial.

- In response to the initial review, the authors state that, in addition to a power analysis targeting main effects, the sample size was increased to account for the detection of interactions. However, this explanation lacks quantitative justification, and it remains unclear whether the current sample size is adequate for the multifactorial analyses actually conducted in this study, including higher-order interactions. In other words, the relationship between the a priori design (power analysis) and the actual analytical strategy remains unclear. The authors should clarify the target effects and underlying assumptions of the power analysis, or alternatively, indicate the range of effect sizes detectable with the current sample size in order to reconstruct the validity of the study design.

- This study employs a multifactorial analysis including a four-way interaction; however, when a higher-order interaction is significant, main effects cannot be interpreted as general tendencies, and the effects of each factor should instead be interpreted as conditional. In the present manuscript, main effect–like descriptions are provided regardless of the presence of interactions, indicating that the principle of hierarchical interpretation is not adequately followed. The authors should clearly distinguish the level at which interpretation is appropriate based on the results of higher-order interactions and restructure the presentation of the results accordingly. In particular, for response time, the manuscript states that when higher-order interactions are significant, the data are decomposed by view (palm vs. back). However, the subsequent analyses and reporting are not consistent with this stated approach. If the data are indeed decomposed by view, analyses should proceed separately within Palm and Back conditions using the remaining factors (laterality, orientation, stimulus type), effectively as three-way ANOVAs, in which case view should no longer appear as an analysis factor. In contrast, interactions involving view continue to be reported, indicating that the analytical procedure is not applied consistently. This inconsistency suggests that the principle of hierarchical interpretation of interactions is not properly maintained.

- Furthermore, although the study assumes that different processing mechanisms underlie palm and back views, it remains unclear how these two conditions are compared and positioned within the analysis. If a direct comparison between palm and back views is not the primary objective, it may be more appropriate to separate the data by view in advance and evaluate the effects of the remaining factors (laterality, orientation, stimulus type) within each view. Indeed, prior studies have adopted such an approach. Therefore, the authors should consider restructuring the analysis as a three-way ANOVA (Laterality × Orientation × Stimulus Type) within each view and interpreting the results accordingly. Clarifying the relationship between the analytical strategy and the research objectives in this manner would help ensure consistency in the interpretation of the findings.

Minor Comments

1. [Abstract] The Abstract is generally clear; however, the results are described in terms of main effects, which may not be consistent with the interaction structure reported in the main text. In particular, the effects of depiction type and view are presented as general tendencies, although they are likely to be condition-dependent. Therefore, the Abstract should be revised to align with the statistical structure of the results.

2. [Introduction] The extrastriate body area (EBA) is introduced; however, it is not sufficiently integrated into the study’s hypotheses or the interpretation of the results. Its role should be clarified or the description should be reorganized.

3. [Participants] The relationship between the number of recruited participants (62) and those who received experimental credit (70) is unclear and may be inconsistent (L.131). It should be clarified whether these refer to the same sample or different populations.

4. [Stimuli and Materials] It is recommended to provide additional details regarding display resolution and stimulus size. Although differences in visual characteristics between depiction types are mentioned in the Discussion, the information provided is insufficient. These details are also important for reproducibility.

5. [Procedure] Although not pointed out in the initial review, the inclusion of a task in training phase 1 that requires participants to physically move their hands to match the stimuli may have induced motor imagery during the main task. As this could affect the interpretation of the results, it is recommended that this possibility be discussed in the Discussion section.

6. [Data processing and analysis – Data exclusion] It is recommended to clarify the rationale for the response time exclusion criteria, either by citing relevant prior studies or by providing a brief justification.

7. [Results - Simple reaction time (SRT)] As this is the Results section, the description should be limited to the presentation of results. The statement that SRT values were not used in subsequent analyses is methodological in nature and should be described in the Methods if necessary. Furthermore, in the absence of statistical testing, the description remains impressionistic and is not appropriate for this section. Any interpretative or suggestive content should instead be addressed in the Discussion.

8. [Figures] The role of the figures in relation to the statistical analyses is unclear. Given that higher-order interactions are reported, figures should be structured to reflect condition-dependent effects rather than general trends. However, the current presentation may give the impression of main effects or overall tendencies, which is not consistent with the reported interaction structure. The authors should clarify the purpose of each figure and ensure that the graphical presentation is aligned with the statistical model and its hierarchical interpretation.

**********

7. PLOS authors have the option to publish the peer review history of their article (what does this mean?). If published, this will include your full peer review and any attached files.

If you choose “no”, your identity will remain anonymous but your review may still be made public.

**Do you want your identity to be public for this peer review?**  For information about this choice, including consent withdrawal, please see our Privacy Policy.

Reviewer #1: No

Reviewer #2: No

**********

[NOTE: If reviewer comments were submitted as an attachment file, they will be attached to this email and accessible via the submission site. Please log into your account, locate the manuscript record, and check for the action link "View Attachments". If this link does not appear, there are no attachment files.]

To ensure your figures meet our technical requirements, please review our figure guidelines: https://journals.plos.org/plosone/s/figures

You may also use PLOS’s free figure tool, NAAS, to help you prepare publication quality figures: https://journals.plos.org/plosone/s/figures#loc-tools-for-figure-preparation.

NAAS will assess whether your figures meet our technical requirements by comparing each figure against our figure specifications.

# Author comment: Author response to Decision Letter 2

**DOI:** 10.1371/journal.pone.0352694.r004

31 May 2026

Please see the attached response letter and revised manuscript. We have carefully addressed all reviewer and editor comments and revised the manuscript accordingly.

Attachment Submitted filename: RevisionLetter_auresp_2.docx

# Peer review: Decision Letter 2

**DOI:** 10.1371/journal.pone.0352694.r005

**Authors:** Giulia Prete, Giulia Prete, Giulia Prete

7 Jun 2026

PONE-D-25-62785R2

The Influence of Hand Depiction Types on Behavioural Patterns in Laterality Judgments

PLOS One

Dear Dr. Saran,

Thank you for submitting your manuscript to PLOS ONE. After careful consideration, we feel that it has merit but does not fully meet PLOS ONE’s publication criteria as it currently stands. Therefore, we invite you to submit a revised version of the manuscript that addresses the points raised during the review process.

==============================

As you can seen, I re-invited the expert who revised the previous version of the manuscript. He/She acknowledges that the manuscript is now improved but also highlighted serious issues. Since the other reviewer had recommended acceptance of the study, I decided not to reject it at this stage. However, I would like to strongly suggest you to fully consider all the present suggestions. Please, pay attention to citations in particular (see Reviewer's comment), as well as to hierarchical interpretation of interaction effects. I will evaluate the next revisions myself before deciding how to proceed.

==============================

Please submit your revised manuscript by Jul 22 2026 11:59PM. If you will need more time than this to complete your revisions, please reply to this message or contact the journal office at plosone@plos.org. When you're ready to submit your revision, log on to https://www.editorialmanager.com/pone/ and select the 'Submissions Needing Revision' folder to locate your manuscript file.

Please include the following items when submitting your revised manuscript:

- A letter that responds to each point raised by the academic editor and reviewer(s). You should upload this letter as a separate file labeled 'Response to Reviewers'.
- A marked-up copy of your manuscript that highlights changes made to the original version. You should upload this as a separate file labeled 'Revised Manuscript with Track Changes'.
- An unmarked version of your revised paper without tracked changes. You should upload this as a separate file labeled 'Manuscript'.

If you would like to make changes to your financial disclosure, please include your updated statement in your cover letter. Guidelines for resubmitting your figure files are available below the reviewer comments at the end of this letter.

If applicable, we recommend that you deposit your laboratory protocols in protocols.io to enhance the reproducibility of your results. Protocols.io assigns your protocol its own identifier (DOI) so that it can be cited independently in the future. For instructions see: https://journals.plos.org/plosone/s/submission-guidelines#loc-laboratory-protocols. Additionally, PLOS ONE offers an option for publishing peer-reviewed Lab Protocol articles, which describe protocols hosted on protocols.io. Read more information on sharing protocols at https://plos.org/protocols?utm_medium=editorial-email&utm_source=authorletters&utm_campaign=protocols.

As the corresponding author, your ORCID iD is verified in the submission system and will appear in the published article. PLOS supports the use of ORCID, and we encourage all coauthors to register for an ORCID iD and use it as well. Please encourage your coauthors to verify their ORCID iD within the submission system before final acceptance, as unverified ORCID iDs will not appear in the published article. *Only*  the individual author can complete the verification step; PLOS staff *cannot*  verify ORCID iDs on behalf of authors.

We look forward to receiving your revised manuscript.

Kind regards,

Giulia Prete

Academic Editor

PLOS One

Journal Requirements:

If the reviewer comments include a recommendation to cite specific previously published works, please review and evaluate these publications to determine whether they are relevant and should be cited. There is no requirement to cite these works unless the editor has indicated otherwise.

[Note: HTML markup is below. Please do not edit.]

Reviewers' comments:

Reviewer's Responses to Questions

**Comments to the Author**

1. If the authors have adequately addressed your comments raised in a previous round of review and you feel that this manuscript is now acceptable for publication, you may indicate that here to bypass the “Comments to the Author” section, enter your conflict of interest statement in the “Confidential to Editor” section, and submit your "Accept" recommendation.

Reviewer #2: (No Response)

**********

2. Is the manuscript technically sound, and do the data support the conclusions?

The manuscript must describe a technically sound piece of scientific research with data that supports the conclusions. Experiments must have been conducted rigorously, with appropriate controls, replication, and sample sizes. The conclusions must be drawn appropriately based on the data presented.

Reviewer #2: No

**********

3. Has the statistical analysis been performed appropriately and rigorously?

Reviewer #2: No

**********

4. Have the authors made all data underlying the findings in their manuscript fully available?

The PLOS Data policy requires authors to make all data underlying the findings described in their manuscript fully available without restriction, with rare exception (please refer to the Data Availability Statement in the manuscript PDF file). The data should be provided as part of the manuscript or its supporting information, or deposited to a public repository. For example, in addition to summary statistics, the data points behind means, medians and variance measures should be available. If there are restrictions on publicly sharing data—e.g. participant privacy or use of data from a third party—those must be specified.

Reviewer #2: Yes

**********

5. Is the manuscript presented in an intelligible fashion and written in standard English?

PLOS ONE does not copyedit accepted manuscripts, so the language in submitted articles must be clear, correct, and unambiguous. Any typographical or grammatical errors should be corrected at revision, so please note any specific errors here.

Reviewer #2: Yes

**********

6. Review Comments to the Author

Please use the space provided to explain your answers to the questions above. You may also include additional comments for the author, including concerns about dual publication, research ethics, or publication ethics. (Please upload your review as an attachment if it exceeds 20,000 characters)

Reviewer #2: The overall organization of the manuscript has improved substantially since the previous revision, and the authors' efforts are appreciated. However, a number of serious problems with the citation of prior literature have been identified. Most critically, one of the references cited in the manuscript (Reference [30]) could not be verified by title, author names, DOI, or direct inspection of the relevant issue of the journal. The citation of a publication that cannot be confirmed to exist raises serious concerns regarding scientific integrity, and makes it difficult to evaluate this manuscript as a scientific paper. Furthermore, numerous instances were found in which the content of cited works does not align with the context in which they are cited. This issue was raised in the previous round of review, and the fact that it remains unresolved after revision suggests a fundamental insufficiency in the authors' understanding and organization of the relevant literature.

The rationale for the research objectives also remains insufficiently developed. It is unclear why this study is necessary and how the proposed objectives follow logically from the existing literature, resulting in a disconnect between the Introduction and the Methods. In addition, simple reaction time (SRT) is measured and reported in the manuscript, yet its purpose is not stated in the Methods, and no interpretation is offered in the Discussion. The failure to account for the significance of a measured variable within the manuscript raises concerns regarding the coherence of the study design.

The statistical procedures have been improved in the revised manuscript. However, the problem of interpreting findings as general tendencies without adequately accounting for the conditional nature of effects indicated by significant interactions remains unresolved, and the hierarchical interpretation of interaction effects cannot be considered appropriate.

**********

7. PLOS authors have the option to publish the peer review history of their article (what does this mean?). If published, this will include your full peer review and any attached files.

If you choose “no”, your identity will remain anonymous but your review may still be made public.

**Do you want your identity to be public for this peer review?**  For information about this choice, including consent withdrawal, please see our Privacy Policy.

Reviewer #2: No

**********

[NOTE: If reviewer comments were submitted as an attachment file, they will be attached to this email and accessible via the submission site. Please log into your account, locate the manuscript record, and check for the action link "View Attachments". If this link does not appear, there are no attachment files.]

To ensure your figures meet our technical requirements, please review our figure guidelines: https://journals.plos.org/plosone/s/figures

You may also use PLOS’s free figure tool, NAAS, to help you prepare publication quality figures: https://journals.plos.org/plosone/s/figures#loc-tools-for-figure-preparation.

NAAS will assess whether your figures meet our technical requirements by comparing each figure against our figure specifications.

# Author comment: Author response to Decision Letter 3

**DOI:** 10.1371/journal.pone.0352694.r006

11 Jun 2026

A revision letter is attached, addressing all reviewers concerns.

Attachment Submitted filename: RevisionLetter_auresp_3.docx

# Editor report: Decision Letter 3

**DOI:** 10.1371/journal.pone.0352694.r007

**Authors:** Giulia Prete, Giulia Prete, Giulia Prete, Giulia Prete

15 Jun 2026

The Influence of Hand Depiction Types on Behavioural Patterns in Laterality Judgments

PONE-D-25-62785R3

Dear Dr. Saran,

We’re pleased to inform you that your manuscript has been judged scientifically suitable for publication and will be formally accepted for publication once it meets all outstanding technical requirements.

Within one week, you’ll receive an e-mail detailing the required amendments. When these have been addressed, you’ll receive a formal acceptance letter and your manuscript will be scheduled for publication.

An invoice will be generated when your article is formally accepted. Please note, if your institution has a publishing partnership with PLOS and your article meets the relevant criteria, all or part of your publication costs will be covered. Please make sure your user information is up-to-date by logging into Editorial Manager at Editorial Manager® and clicking the ‘Update My Information' link at the top of the page. For questions related to billing, please contact billing support.

If your institution or institutions have a press office, please notify them about your upcoming paper to help maximize its impact. If they’ll be preparing press materials, please inform our press team as soon as possible -- no later than 48 hours after receiving the formal acceptance. Your manuscript will remain under strict press embargo until 2 pm Eastern Time on the date of publication. For more information, please contact onepress@plos.org.

Kind regards,

Giulia Prete

Academic Editor

PLOS One

Additional Editor Comments (optional):

I have carefully read the revised version of the manuscript and believe you have addressed the issues raised by the reviewer in the previous review phase. I would like to thank you and, especially, the reviewers for their valuable contributions to the revision of this manuscript.

# Editor report: Acceptance letter

**DOI:** 10.1371/journal.pone.0352694.r008

**Authors:** Giulia Prete, Giulia Prete, Giulia Prete, Giulia Prete

PONE-D-25-62785R3

PLOS One

Dear Dr. Saran,

I'm pleased to inform you that your manuscript has been deemed suitable for publication in PLOS One. Congratulations! Your manuscript is now being handed over to our production team.

At this stage, our production department will prepare your paper for publication. This includes ensuring the following:

* All references, tables, and figures are properly cited

* All relevant supporting information is included in the manuscript submission,

* There are no issues that prevent the paper from being properly typeset

You will receive further instructions from the production team, including instructions on how to review your proof when it is ready. Please keep in mind that we are working through a large volume of accepted articles, so please give us a few days to review your paper and let you know the next and final steps.

Lastly, if your institution or institutions have a press office, please let them know about your upcoming paper now to help maximize its impact. If they'll be preparing press materials, please inform our press team within the next 48 hours. Your manuscript will remain under strict press embargo until 2 pm Eastern Time on the date of publication. For more information, please contact onepress@plos.org.

You will receive an invoice from PLOS for your publication fee after your manuscript has reached the completed accept phase. If you receive an email requesting payment before acceptance or for any other service, this may be a phishing scheme. Learn how to identify phishing emails and protect your accounts at https://explore.plos.org/phishing.

If we can help with anything else, please email us at customercare@plos.org.

Thank you for submitting your work to PLOS ONE and supporting open access.

Kind regards,

PLOS ONE Editorial Office Staff

on behalf of

Dr. Giulia Prete

Academic Editor

PLOS One
